# Binding and Blocking of Influenza A WT and S31N M2 by Cage Alkyl Amine-Containing Inhibitors

**DOI:** 10.1021/acsomega.6c06359

**Published:** 2026-10-23

**Authors:** Marianna Stampolaki, Kumar Tekwani Movellan, Kyriakos Georgiou, Abel Cherian Varkey, Andreea L. Turcu, Santiago Vázquez, Stefan Becker, Antonios Kolocouris, Loren B. Andreas

**Affiliations:** † Department of NMR-Based Structural Biology, 28282 Max Planck Institute for Multidisciplinary Sciences, Am Fassberg 11, Göttingen 37077, Germany; ‡ Laboratory of Medicinal Chemistry, Section of Pharmaceutical Chemistry, Department of Pharmacy, 69232 National and Kapodistrian University of Athens, Panepistimiopolis-Zografou, Athens 15771, Greece; § Department of Chemistry and Biochemistry, University of Delaware, Newark, Delaware 19716, United States; ∥ Laboratori de Química Farmacèutica, Facultat de Farmàcia i Ciències de l’Alimentació, 16724 Universitat de Barcelona, Barcelona 08028, Spain; ⊥ Institute of Biomedicine of the University of Barcelona, IBUB, Barcelona 08028, Spain

## Abstract

Proton conductance of the wild-type (WT) M2 protein of influenza A is inhibited by cage alkyl amines, which bind in the pore of the channel. Conjugates with a methylene-linked aryl group have been shown to block the channel of the prevalent S31N strain with a different mechanism, but at the same binding site. Here, we investigated the binding and blocking of WT and S31N M2 by a set of these amines using different biophysical methods. The binding behavior of the ligands was explored using magic angle spinning NMR. NMR clearly detected binding for WT but not S31N. Correlation of binding data with ligand cage size suggested a significant structural barrier. Using a liposomal proton flux assay, we confirmed that the compounds identified as binders by NMR also blocked the channel. To assess the binding mode of these small molecules, molecular dynamics simulations were performed. The simulations predicted different binding modes to WT or S31N. No binding to the S31N variant was detected by NMR, nor was the channel effectively blocked in the liposomal proton flux assay or MD simulations. A comparison of M2_18–60_ with ectodomain-truncated M2 (M2_21–97_) suggests that a substantial structural barrier limits entry of bulky cage amines and that the structural barrier is influenced by the presence or absence of cytoplasmic residues.

## Graphical abstract

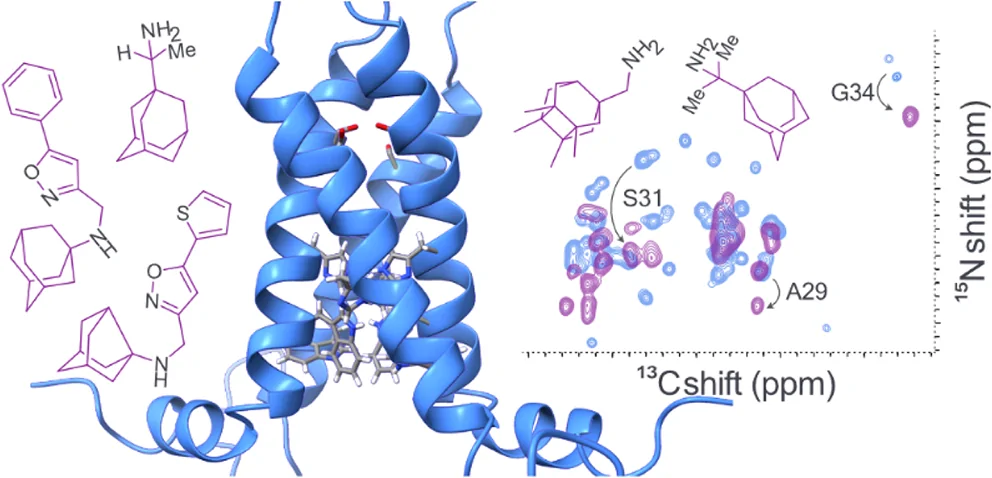

## Introduction

The influenza A matrix 2 (M2) protein is a 97-residue single-pass homotetrameric membrane protein. It contains an extracellular N-terminal domain (residues 21–24), which is highly conserved but not structured and is suggested to assist M2 incorporation into the virion.^1^ A single helical transmembrane (TM) domain (residues 25–46) follows, along with an amphipathic helix (residues 47–60) and a C-terminal intracellular tail (residues 61–97) is known to interact with the M1 protein.^2^ Influenza M2 protein plays a key role during the early and late stages of the virus life cycle via dual function of proton conduction and membrane deformation. The M2 protein forms a membrane-spanning acid-activated proton selective channel.^3,4^ When the virus enters and infects a cell by endocytosis, the influenza M2 channel is activated in response to the lower pH of the endosome,^5^ resulting in proton transport into the virus interior, which triggers the release of the viral RNA^6^ and replication by the host cell.^7^ In a later stage of virus infection, the M2 protein maintains a relatively high pH in the trans-Golgi network and thus prevents premature conformational changes in hemagglutinin (HA) that occur at low pH to trigger membrane fusion.^8^ M2 protein also affects plasma membrane deformation and membrane scission, through its amphipathic helix (AH), which begins at residue 47 and includes up to residues 60, 61, or 62 in reconstituted samples. The AH is needed for virus budding.^9–11^ The TM together with the AH form the functional core of M2, which is called the M2 conductance domain (M2CD^12^) since it precisely reproduces the proton transport properties of full-length M2 (M2FL).^13^ Four His37 residues, known to be the heart of the proton conducting channel, reside near the center of the TM helix and are the key to the proton transport mechanism.^14^

Amantadine (Amt),^15^ rimantadine (Rmt),^16,17^ and other adamantyl,^18–22^ or cage amine analogs,^23–26^ inhibited proton conductance of the wild-type (WT) M2 channel, bearing serine at position 31, as detected by electrophysiology (EP) experiments.^27^ However, due to the emergence of Amt-resistant viruses with mutations in the M2 transmembrane domain including L26F, V27A and the prevalent S31N variant, Amt and Rmt drug usage has been abrogated. To counter resistance, a second generation of adamantane-based drugs, which are methylene-linked aryl conjugates of amantadine, have been synthesized and found to block S31N M2 channel-mediated proton current by EP.^27–31^

Extensive high-resolution structural studies have been conducted on both WT and S31N M2, which were reconstituted in different detergent and lipid environments. These studies utilized solid-state NMR (ssNMR) in phospholipid bilayers,^32–40^ as well as solution NMR in micelles^12,29,41,42^ and X-ray crystallography using detergents^42–44^ or lipidic cubic phase.^45,46^ These reports encompassed protein sequences of varying lengths, strain, WT or mutant M2 sequences, and measurements conducted both with and without bound adamantane-based drugs. As a result, a diverse range of structural observations have been reported.

One such observation is the formation of hydrogen bonds between His37 residues. Initially, such an interaction was proposed to take place for His-His^+^ pairs based on nitrogen and proton chemical shifts.^14,35,47^ Conduction was proposed to take place via protonation of one His-His^+^ pair to form a +3 state for the His tetrad prior to deprotonation and reformation of the +2 state of the His tetrad.^14,35,47^ Measurement of a J-coupling across the histidine residues provided further evidence for a hydrogen bond.^48^ We also observed such a hydrogen bond interaction via a J-coupling, but at high pH for the neutral charge state of the channel (where His37 tetrad has +0 charge)^49^ using M2CD. The CD construct (M2_18–60_) assembles as a dimer-of-dimers in lipid bilayers, consistent with symmetry breaking via the hydrogen bond interaction at His37.^33,50–52^

Structural studies have also reached a consensus that adamantyl amines, e.g., Amt and Rmt, inhibit WT M2-mediated proton conductance by binding to the pore with their ammonium group pointing toward the His37 tetrad and the adamantane group being accommodated by the Val27 tetrad, according to X-ray crystallography^17,44,53^ and NMR with WT M2TM^38,54^ or M2CD.^39,55^ Furthermore, conjugates of amantadine with an aryl group also inhibit S31N M2-mediated proton conductance by occlusion of the pore with their ammonium groups binding to asparagine-31 side chains and the aryl group clustering with Val27 side chains, while the adamantane group is packed in between Ala30 and Gly34, as was shown using solution NMR,^29,56,57^ and MD simulations.^27,29,56^ In the NMR studies, ^13^C–^2^H distance measurements of the binding of adamantane-based derivatives to WT M2TM and M2CD^39^ or ^15^N–^13^C distance measurements to WT^55^ and S31N M2CD^57^ consistently located drug molecules within the pore of M2.

Although both adamantyl amines and aryl conjugates of amantadine occlude the M2 pore, only the adamantyl amines have been proposed as “mechanism-based” inhibitors in the case of WT M2. That means that their ammonium groups bind to water-lined sites at Gly34 or Ala30 of WT M2 that are hypothesized to stabilize transient hydronium ions bound to His37. The hydronium ions that interact with His37 are formed in the proton-conduction mechanism according to X-ray crystallography^17,44,53^ and multiscale MD simulations.^58^ In the drug-bound state, the p*K*_a_ of His37 is lower, suggesting that the stabilized hydronium ions may be in electrostatic communication with the His37 residues.^59^ Note that this proposed mechanism is agnostic concerning the presence of His-His hydrogen bonds, which were observed in the NMR studies,^14,48,49^ but not by crystallography.

Drug binding is commonly inferred through NMR experiments using chemical shift perturbations (CSPs) for pore-lining residues.^18,22,39,57,60,61^ These measurements are sensitive to both direct effects of binding, as well as indirect conformational changes in M2, which may vary depending on the M2 construct and the phospholipid bilayer being used. For example, bound Amt was detected in M2CD (M2_21–61_) reconstituted in 1,2-dimyristoyl-*sn*-glycero-3-phosphocholine (DMPC) bilayers. Yet when a virus-mimetic membrane containing 30% cholesterol was used, no ^15^N or ^13^C CSPs were observed for pore-lining residues, whereas M2TM (M2_22–46_) in the same membrane exhibited drug-induced chemical shift changes.^39^ These observations showed that the energetics of drug binding interactions may considerably differ depending on the sample conditions, including the composition of the membrane and the presence or absence of cytosolic residues. We recently observed slow kinetics of drug binding for M2CD (M2_18–60_), which are modulated by the lipid composition and pH, and can kinetically trap the sample in the pore-unbound state.^50^ Such insights help to understand the lack of binding observed previously for M2CD (M2_21–61_).^39^

Scheme 1 shows a selection of previously synthesized compounds reported to inhibit WT, S31N, or both: the adamantyl amines Rmt, AK59,^19^ a cage alkyl amine RL208,^62^ and aryl conjugates of amantadine M2WJ352 ^29,57^ and UBALT09.^63^ AK59 has one additional methyl group compared to Rmt, while RL208 (3,4,8,9-tetramethyltetracyclo­[4.4.0.0^3,9^.0^4,8^]­dec-1-yl)­methylamine) bears a tetracyclic alkyl cage isomeric to the adamantyl group but is larger in size than Amt. The conjugate M2WJ352 is a prototype selective S31N M2 blocker by EP. Its analog, UBALT09, replaces adamantane with 3-noradamantane, which is a minimal reduction in size. It is also a selective blocker of S31N M2 by EP.

**Scheme 1:**
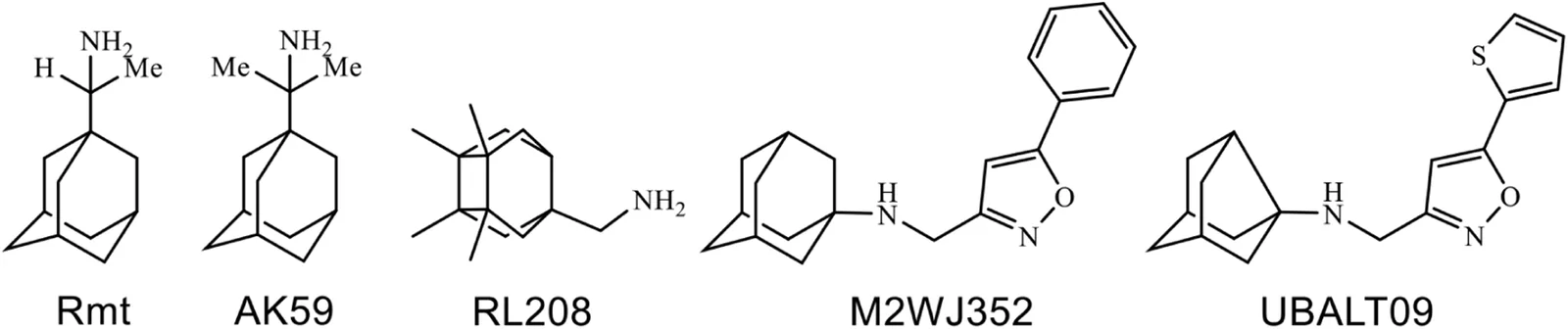
Chemical Structures of Rmt, AK59, RL208, M2WJ352, and UBALT09 Tested against WT and S31N M2_18‑60_

The inhibition of proton conductance was previously investigated via the Two-Electrode Voltage-Clamp assay (TEVC). The blocking efficiency of Rmt,^16,17^ AK59,^19^ and RL208 ^60^ was assessed by EP against M2FL at 2 min of 100 μΜ drug exposure using the TEVC assay. All cage alkyl amines Rmt, AK59, and RL208 block WT M2-mediated proton current but not S31N M2 by EP. RL208 is a triple blocker of WT, L26F and V27A M2FL protein channels by EP,^60^ and M2WJ352 and UBALT09 selectively inhibited S31N.^29,57,63^ This previously reported blocking activity by EP against the M2FL is included in Table S1.

In previous work^49,50^ we observed disruption of the N–H–N hydrogen bond between imidazoles of His37 residues in adjacent TM helices upon Rmt binding to WT M2CD (M2_18–60_) using magic angle spinning (MAS) NMR. This was consistent for several bilayer preparations including 1,2-diphytanoyl-sn-glycero-3-phosphocholine (DPhPC), DPhPC with cholesterol, and in a virus-mimetic membrane. This finding is consistent with a mechanism-based inhibition in which the drug binding perturbs the His37 structure, or disrupts the structure of functional water molecules, as was previously suggested.^44,58^ The disruption of the His37 N–H–N hydrogen bond is significant since it has a straightforward association with the inhibition mechanism of the WT M2 proton conductance inside the pore by adamantyl amines.

To expand on this mechanism-based inhibition hypothesis, based on our previous NMR findings,^49,50^ in this work we compared the binding of Rmt and four previously synthesized compounds (Scheme 1) to M2, using MAS NMR, EP, a liposomal proton flux assay, and MD simulations.

## Results

### Magic Angle Spinning NMR

#### Drug Binding to M2CD Tetramer at Elevated Temperatures

We recorded proton-detected (H)­NH NMR spectra of M2CD (M2_18–60_) reconstituted in perdeuterated DPhPC membranes. The lipid to tetramer molar ratio was 25:1 which favors sensitivity due to a high concentration of tetrameric M2CD. The site-specific resolution allows tracking of Gly34 backbone and His37 side chain resonances that are strongly affected by drug binding to the pore. Thus, the binding was tracked primarily by measuring changes in the chemical shifts of ^15^N and ^1^H nuclei in Gly34 or His37. A solution of 300 μΜ of drug was equilibrated with M2CD embedded in DPhPC lipid at 4 °C. When lack of binding occurred, the sample was held at elevated temperature, at 55 °C for 2–5 h in the case of WT M2CD and for up to >10 h at 70 °C in the case of S31N M2CD, which helps to overcome the high energy barrier to drug entry.^50^ Table S2 summarizes the CSPs observed in the spectra. It is important to note that all the spectra were recorded at room temperature and the fact that no CSPs are observed after incubation at an elevated temperature without the presence of a drug indicates that the protein structure is stable at these elevated temperatures. In addition, residue peaks appear in duplicate in the labeled 2D or 3D spectra. This doubling is the result of the 2-fold symmetric structure (dimer-of-dimers) that has inequivalent resonances for peptide chains “A” and “B” of the tetramer.

##### WT M2CD

As shown in Figure 1C–E, we did not observe significant CSPs of WT M2CD protein resonances in the presence of the tested compounds Rmt, AK59, and RL208 at 4 °C. For Rmt, changes in the chemical shifts were smaller than 0.5 ppm for ^13^C and ^15^N and smaller than 0.2 ppm for ^1^H nuclei. These CSPs do not surpass the value of 0.2 ppm calculated as $\surd \frac{1}{3}\left[\Delta \delta_{Η}^{2}+0.14\Delta \delta_{N}^{2}+0.3\Delta \delta_{C}^{2}\right]$ ^64^ . These changes occur due to nonspecific effects, rather than pore binding since entry to the pore is exceedingly slow under these conditions.^32,50^

**Figure 1:**
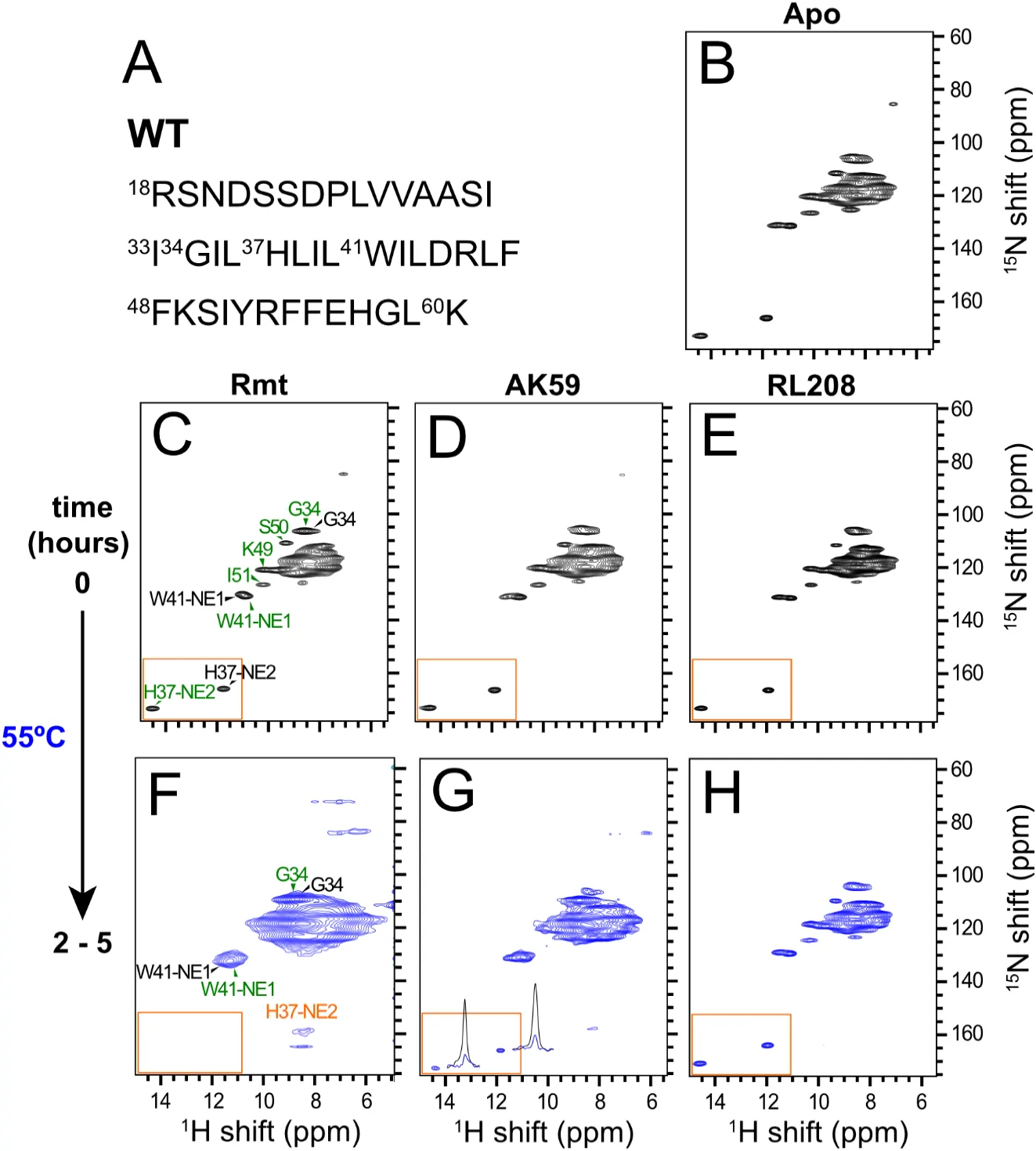
Monitoring the ^1^H and ^15^N CSPs in WT M2CD spectra in the presence of small molecules. (A) shows the amino acid sequence of M2CD; (B) shows the (H)­NH spectrum of WT M2CD (without ligand) measured at room temperature; (C)–(E) show the (H)­NH spectra of WT M2CD with Rmt or AK59 or RL208, respectively, incubated at 4 °C; (F)–(H) show the (H)­NH spectra of WT M2CD with Rmt or AK59 or RL208, respectively, after 2–5 h of incubation at 55 °C, and the His37 side chain peak intensities for selected spectra. The samples were reconstituted in DPhPC bilayers at pH 7.8 using a lipid:tetramer molar ratio of 25:1, and incubated with 300 μM drug. Residue labels of the dimer-of-dimers structure are colored black for chain A and green for chain B.

After incubation at a higher temperature of 55 °C, large shifts across the whole protein sequence were induced by Rmt for WT M2CD. Some of those shifts have values greater than 2 ppm, e.g., ca. 2 ppm in the ^15^N resonance of Gly34 and >4 ppm in the His37-Nε2 side chain and ca. 5 ppm in the ^1^H resonance of His37-Nε2 side chain (Table S2, Figures 1F, 3, 4), suggesting, as previously reported, significant restructuring of the protein,^32,49,50^ during pore-binding.^39^ One clear structural change that occurs upon pore binding by Rmt is the disruption of the imidazole-imidazole hydrogen bonding between His37 residues in adjacent TM helices indicated by the absence of the proton resonance at 14.3 and 11.7 ppm^49^ (Figure 1F). This is also evident for AK59, but the remaining intensity of His37-Nε2 side chain peaks at 14.3 and 11.7 ppm or of Gly34 in the (H)­NH spectrum after 5 h at 55 °C (Figure 1G) suggested the presence of two populations, the bound and unbound form of M2CD. From measuring the intensity of the His37-Nε2 side chain peaks, ca. 20% of the population corresponds to the unbound protein, based on the ∼80% disruption of the hydrogen bonding (∼20% of the remaining intensity of His37-Nε2 peaks). The CSPs of AK59 binding were similar in magnitude to Rmt binding. Specifically, the changes in the chemical shifts in the ^15^N resonance of Gly34 and ^15^N and ^1^H resonances of His37-Nε2 were >4 ppm. We observed no binding for RL208 at the elevated temperature (Figure 1H).

##### S31N M2CD

In the (H)­NH spectra of S31N M2CD, none of the tested compounds showed any significant effect on the imidazole-imidazole hydrogen bond between His37 residues or other residues in S31N M2 (Figure 2) which was unexpected for M2WJ352 and UBALT09 that block the full-length S31N M2 channel in EP experiments^29^ (Table S1).

**Figure 2:**
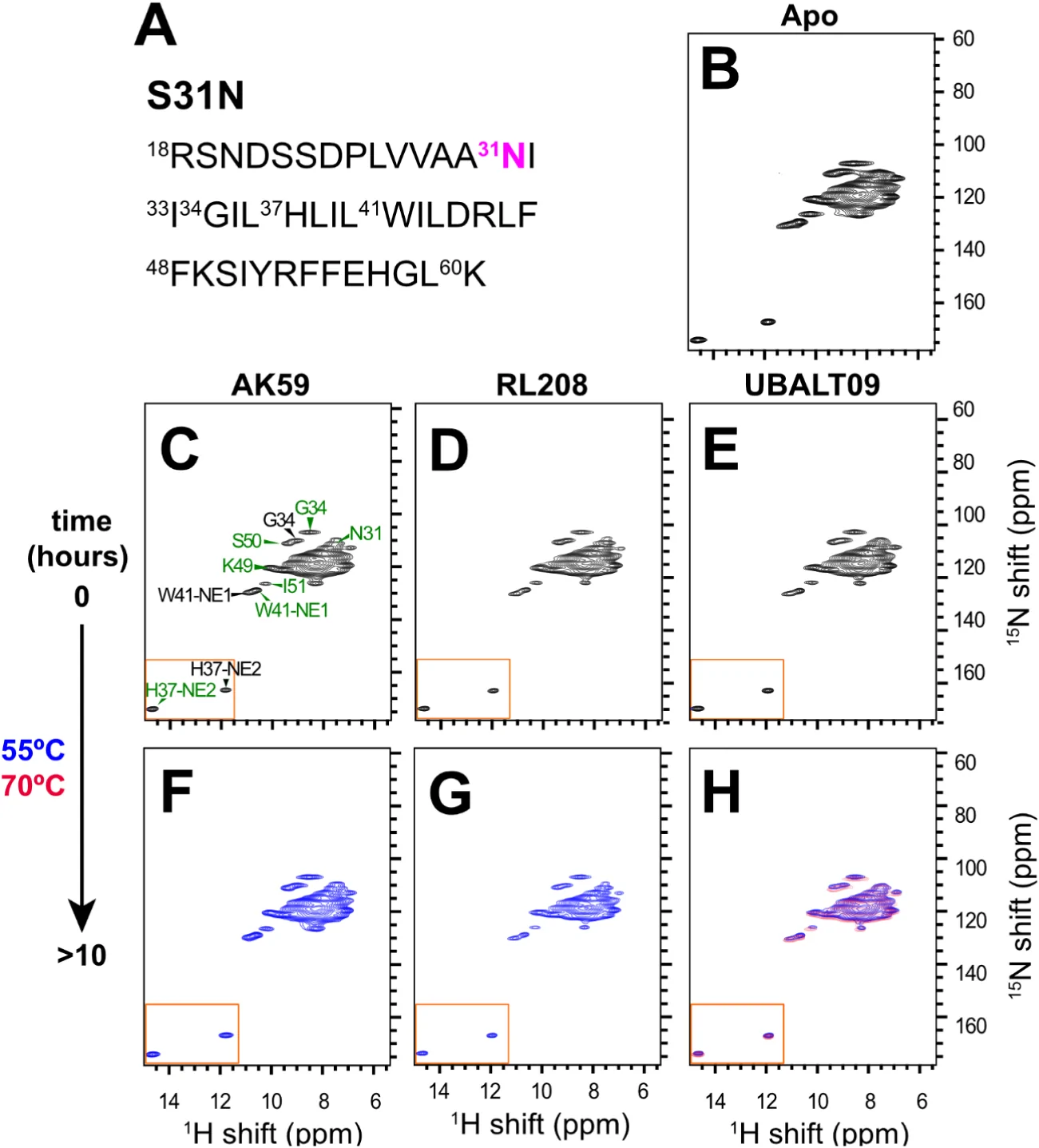
Monitoring the ^1^H and ^15^N CSPs from S31N M2CD spectra in the presence of small molecules. (A) shows the amino acid sequence of the construct; (B) shows the (H)­NH spectrum of S31N M2CD (without ligand) measured at room temperature; (C)–(E) show (H)­NH spectra of M2CD with AK59 or RL208 or UBALT09, incubated at 4 °C, respectively. (F)–(H) show the (H)­NH spectra of S31N M2CD with AK59 or RL208 or UBALT09, after >10 h of incubation at 55 °C (70 °C for UBALT09 in red), respectively. The samples were reconstituted in DPhPC bilayers at pH 7.8 using a lipid:tetramer molar ratio of 25:1, and incubated with 300 μM drug. Residue labels of the dimer-of-dimers structure are colored black for chain A and green for chain B.

#### Drug Binding to M2CD Tetramer at Low pH

Based on the reported p*K*_a_ values,^14,33,59,65^ at the acidic pH of 3.5 the imidazoles of the four His37 become protonated, and M2 adopts an open conformation at the C-terminal end in which His37-His37 hydrogen bonding is disrupted. A more open conformation is consistent with crystallography data^43,45^ and with a large change in the measured TM helical tilt in WT M2CD.^66^ Following this data, we adopted a protocol, previously applied to M2TM (M2_22–46_),^67^ by first shifting the M2CD protein sample to pH 3.5 and incubating overnight in the presence of the drug at 4 °C, and then shifting the pH back to 7.8 for NMR-based analysis of CSPs, still in the presence of the drug. The spectrum was then measured at pH 7.8 at room temperature.

Spectral changes induced by low pH are reversible in the absence of drug. Figures S1A, B and S2A show the relevant spectra for the apo WT or S31N M2CD, respectively. Starting with the protein sample at pH 7.8, the (H)­NH spectra showed the His37-Nε2 proton resonances at 14.3 and 11.7 ppm, which are characteristic of the imidazole-imidazole hydrogen bonded state.^49,50^ After overnight incubation at pH 3.5, we observed the expected His37-His37 hydrogen bonding disruption for both WT and S31N M2CD (Figures S1B, S2B). After incubating the protein at pH 7.8 again, the peaks characteristic of the imidazole-imidazole hydrogen bond returned in the spectra of apo WT or S31N M2CD (Figures S1C, S2C).

##### WT M2CD

Figures 3 and 4 show WT M2CD spectra demonstrating nearly complete drug binding using the pH shift protocol, with measurement at pH 7.8 for both Rmt,^49,50^ and AK59. As shown in Figures 3A and 4A we observed the clear disruption of the His37-His37 hydrogen bond in the presence of Rmt or its analog AK59. For the latter, only 0.5% population was left unbound, based on the His37 peaks. Other distinct CSPs that revealed binding are those of well-separated peaks, for example Gly34, Lys49, Ser50, Ile51 backbone, and Trp41 side chains. The rest of the residues in the (H)­NH spectrum was not resolved, which is not surprising since the protein was not deuterated. To obtain spectra with increased resolution, we recorded 3D (H)­CANH spectra that turned out to be very similar for Rmt and AK59, showing large CSPs along the whole transmembrane helix, as well as the characteristic disappearance of signals from the amphipathic helix. The ^15^N–^13^Cα projections of the (Η)­CANH spectrum for Rmt and AK59 samples are shown in Figures 3B and 4B, respectively. An H^N^, N, Cα CSP analysis was carried out for each detected residue as described previously^64^ and is shown in Figure S9. Compound RL208 at 300 μΜ concentration did not disrupt the His37-His37 hydrogen bond, the amide region in the 2D (H)­NH spectrum showed only minor broadening, and the ^15^N–^13^Cα spectrum was unchanged from the spectrum of the apo protein^60^ (Figure S1C). When we increased the drug concentration to 6 mM, RL208 still did not disrupt His37-His37 hydrogen bonding although it produced broadening in the main peak region of the (H)­NH spectrum and chemical shift changes of 0.5 and 1.5 ppm in ^1^H and ^15^N resonances, respectively, of one of the Trp41-NE1 side chain peaks (Figure S3). There were also minor chemical shift changes in the well-separated peaks from Gly34, Lys49, Ser50, and Ile51. In the 3D (H)­CANH spectrum the chemical shift changes from the better resolved peaks did not exceed 0.5 ppm in ^15^N and ^13^C resonances (Figure S3).

**Figure 3:**
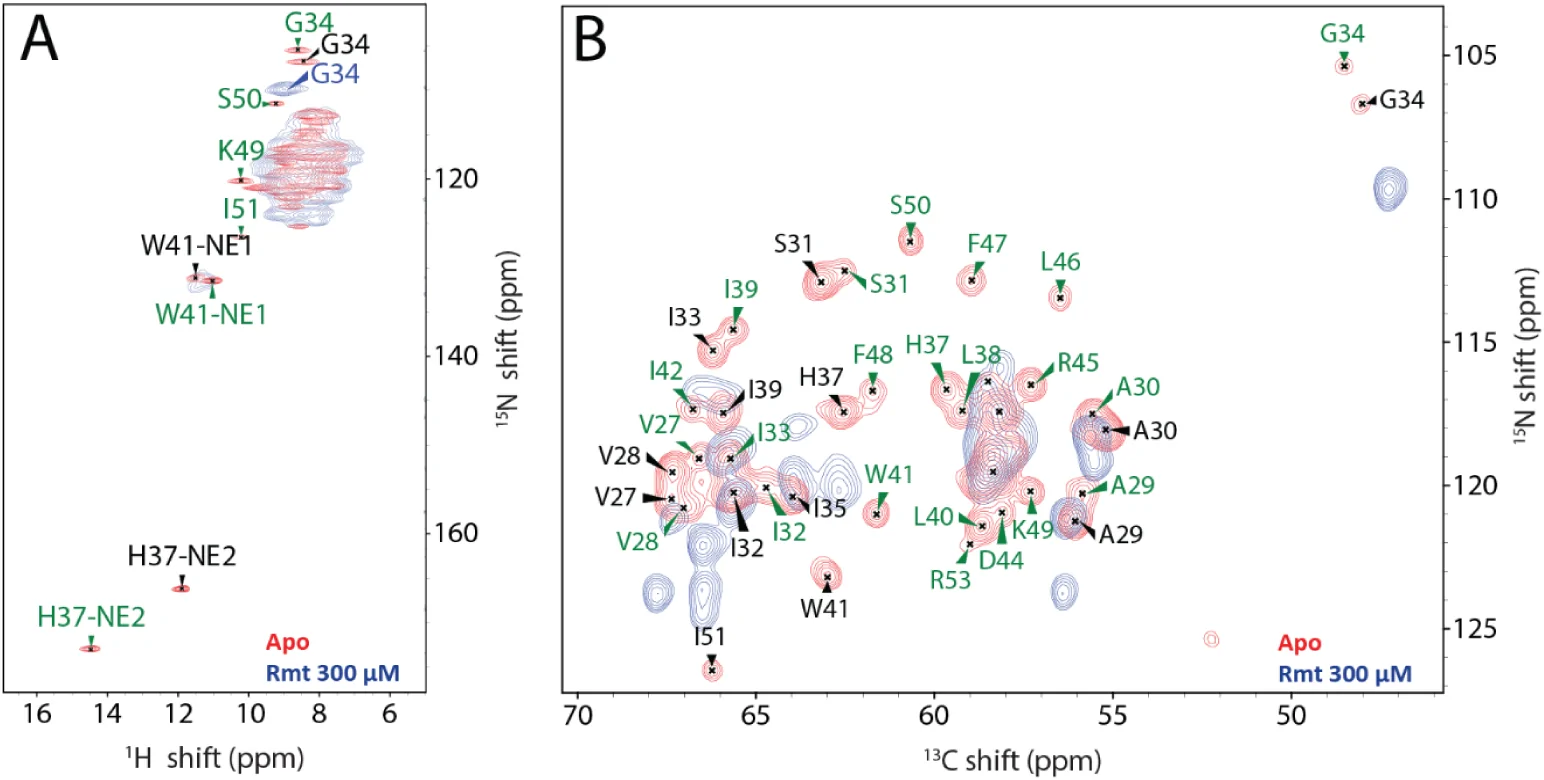
Monitoring the ^1^H, ^15^N, and ^13^C CSPs in WT M2CD resonances in the presence of 300 μM Rmt. (A) (Η)­NH spectrum of WT M2 with (blue) and without Rmt (red). (B) ^15^N–^13^Cα projection of the (Η)­CΑNH spectrum of WT M2 with (blue) and without Rmt (red). The samples were reconstituted in DPhPC bilayers at pH 7.8 using a lipid:tetramer molar ratio of 25:1. Both spectra were recorded at pH 7.8 after incubating the system at pH 3.5 in the presence of the drug and switching back to pH 7.8. Residue labels of the dimer-of-dimers structure are colored black for chain A and green for chain B.

**Figure 4:**
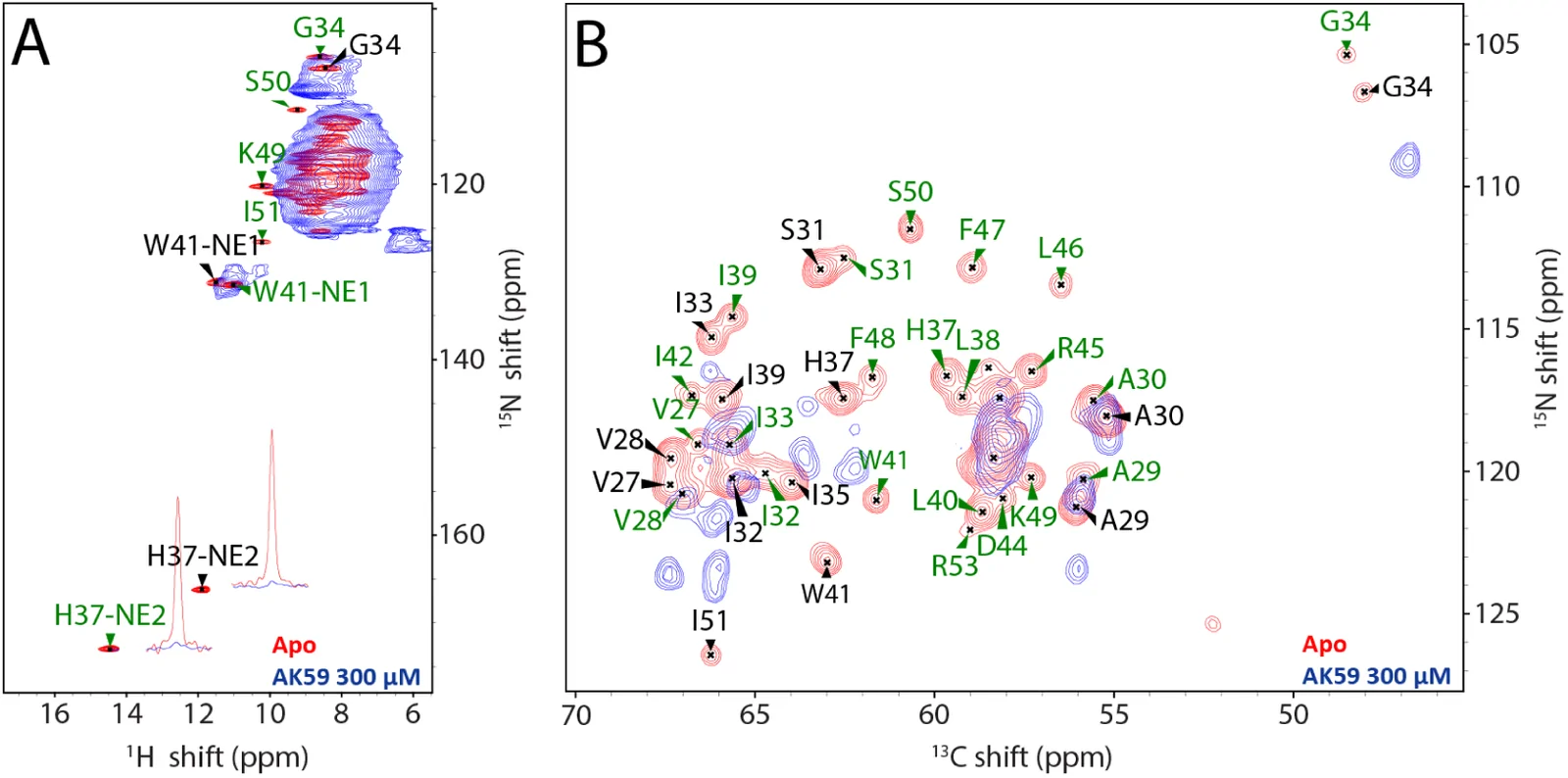
Monitoring the ^1^H, ^15^N, and ^13^C CSPs in WT M2CD resonances in the presence of 300 μM AK59. (A) (Η)­NH spectrum of WT M2 with (blue) and without AK59 (red) and His37 side chain peak intensities. (B) ^15^N–^13^Cα projection of the (Η)­CANH spectrum of WT M2 with (blue) and without AK59 (red). The samples were reconstituted in DPhPC bilayers at pH 7.8 using a lipid:tetramer molar ratio of 25:1. Both spectra were recorded at pH 7.8 after incubating the system at pH 3.5 in the presence of the drug and switching back to pH 7.8. Residue labels of the dimer-of-dimers structure are colored black for chain A and green for chain B.

In the case of UBALT09, which is not a blocker of the WT M2 channel by EP (Table S1), using a drug concentration of 300 μΜ we did not observe His37-His37 hydrogen bonding disruption (Figure 5). Interestingly, the (H)­NH spectrum showed doubling of the His37-Nε peaks and a broadening of the main peak region (Figure 5A, upper part) as well as chemical shift perturbation of 0.5 and 1.5 ppm in ^1^H and ^15^N resonances, respectively, of one of the Trp41-Nε side chains. However, in the (H)­CANH spectra no doubling of peaks or other significant chemical shift changes are evident. We incubated the sample in a higher drug concentration of 6 mM. The results are shown in Figure 5, where in the (H)­NH spectrum we observed ∼30% of the His37-Nε2 side chain peak intensity remaining, as well as a chemical shift change in the ^15^N resonance of Trp41 side chain, and a general broadening of the amide region of the (H)­NH spectrum. Additionally, in the (H)­CANH spectrum we observed large chemical shift changes but also peaks which revealed two populations, which we assign to the apo-form and drug-bound M2 (Figure 5).

**Figure 5:**
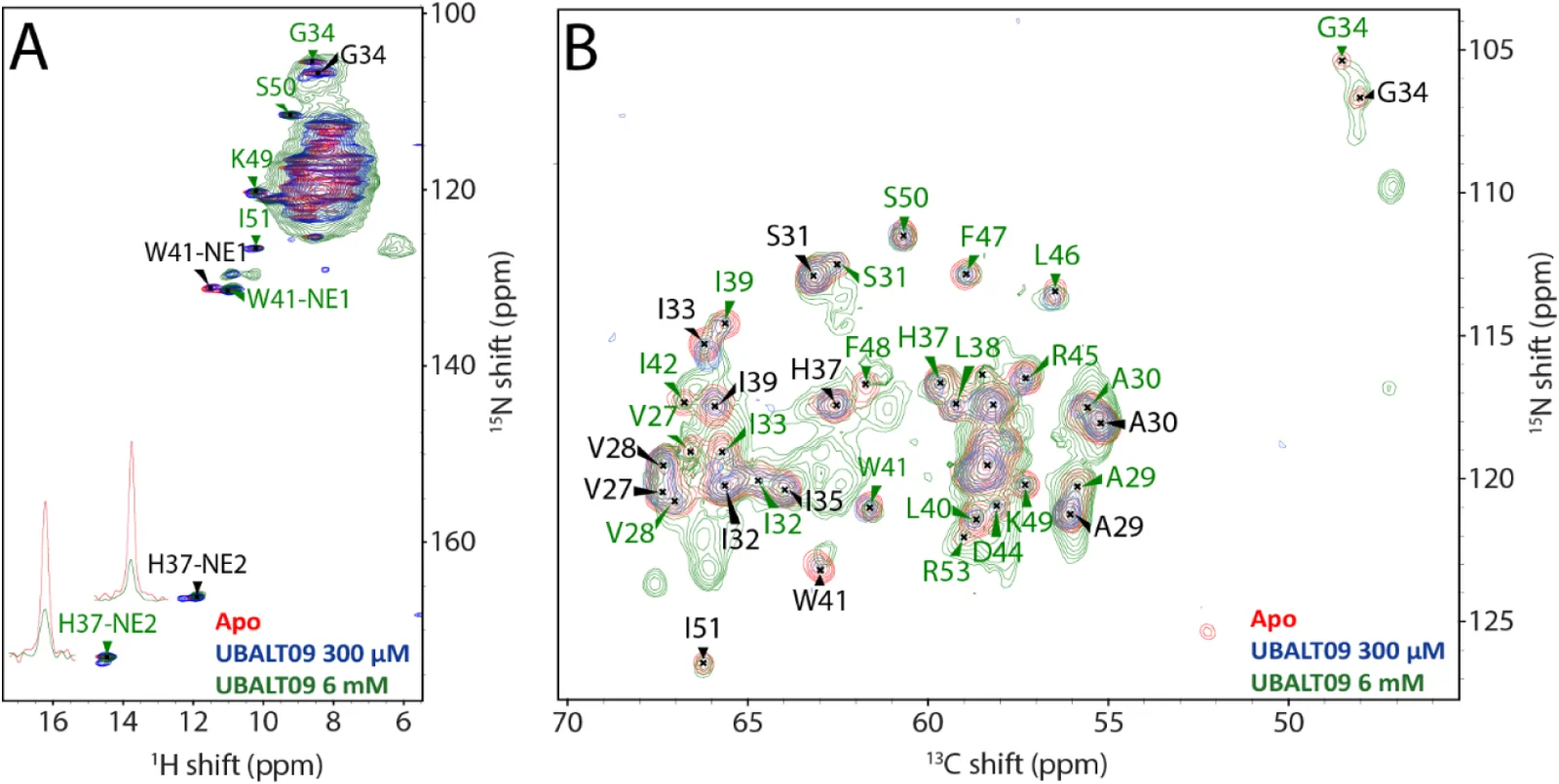
Monitoring the ^1^H, ^15^N, and ^13^C CSPs in WT M2CD resonances in the presence of 300 μΜ or 6 mM UBALT09. (A) (Η)­NH spectrum of WT M2 with UBALT09 at 300 μM (blue), or 6 mM (green) and without UBALT09 (red) and His37 side chain peak intensities. (B) ^15^N–^13^Cα projection of the (Η)­CANH spectrum of WT M2 with UBALT09 at 300 μΜ (blue), or 6 mM (green) and without UBALT09 (red). The samples were reconstituted in DPhPC bilayers at pH 7.8 using a lipid:tetramer molar ratio of 25:1. Both spectra were recorded at pH 7.8 after incubating the system at pH 3.5 in the presence of the drug and switching back to pH 7.8. Residue labels of the dimer-of-dimers structure are colored black for chain A and green for chain B.

##### S31N M2CD

Regardless of the incubation protocol, no NMR-based evidence of drug binding was found for S31N M2CD with Rmt, AK59, or RL208. However, M2WJ352 and UBALT09 were efficient blockers of full-length S31N M2 in the EP assay (Table S1). In the samples of all drugs, we observed the His37-Nε2 proton resonances at 14.3 and 11.7 ppm and we did not observe CSPs in the 2D or 3D spectra (Figure S2C), i.e., the spectra from all the tested drugs were almost identical to the spectrum of the apo S31N M2. When we increased the drug concentration to 6 mM for M2WJ352 and UBALT09 His37-His37 hydrogen bonding was again maintained but some small chemical shift changes were observed. In particular, at Gly34 they were not larger than 0.5 ppm in ^15^N or ^13^C resonances in both 2D and 3D spectra (Figures S5, S6). Those CSPs are likely due to the partitioning of the drug to the membrane^39,68^ as well as potential peripheral binding to the protein^12,55^ and are of similar magnitude as was previously observed for Rmt^50^ and characterized as nonspecific binding.

#### MD Simulations

We carried out 250 ns-MD simulations of WT or S31N M2CD with or without drugs inside the M2 pore. We simulated the Rmt enantiomers (R-Rim, S-Rim), AK59, RL208, M2WJ352, and UBALT09 in complex with WT or S31N M2CD embedded in POPC bilayers using the CHARMM36m force field.^69,70^ Although POPC bilayers were chosen for computational convenience (well characterized bilayer, well-optimized force fields), we carried out simulations for selective blockers using DPhPC bilayers to be consistent with the NMR sample environment. We then calculated the pore radius of M2CD in the two different lipids as a measure of potential changes in the packing of the tetramer. The results showed that there are only very slight differences in the range of 0.0–0.3 Å (Table S5). Additionally, we extended the simulations to protonated or nonprotonated His37. Altogether, we monitored the water traversal through the pore as a way of assessing inhibition. It should be clear that the blockage of water passage mostly resembles pore occlusion mechanism rather than “mechanism-based” inhibition, as the formation of hydronium ions cannot be represented in MD simulations. Still, in the case of WT inhibitors, we detect stabilization of water clathrate between the ligand’s ammonium group and the histidines.

##### WT M2CD

We generated docking poses of the adamantyl amine drugs, RL208, or M2WJ352, and UBALT09 inside WT M2CD having their ammonium group oriented toward the C-terminus (inward) as suggested from experiments and MD simulations of complexes between Amt or Rmt or other inhibitors and M2 using different WT or S31N constructs.^17,44,54^ Our MD simulations of adamantyl amines (R-Rim, S-Rim, AK59) and RL208 in complex with the WT M2CD showed that the cage alkyl amines form ∼3 hydrogen bonds with waters between the ligand and His37 (Figure 6, Tables S3, S4). Compared to the apo WT M2CD, where a free flow of waters from the Val27 secondary gate to Trp41 was observed (Figure S11), in these complexes almost no waters passed from the Val27 gate (see also SI for complementary results). In contrast, in the complexes with M2WJ352 and UBALT09, waters were able to pass the Val27 secondary gate (Figure 6, Table S7).

**Figure 6:**
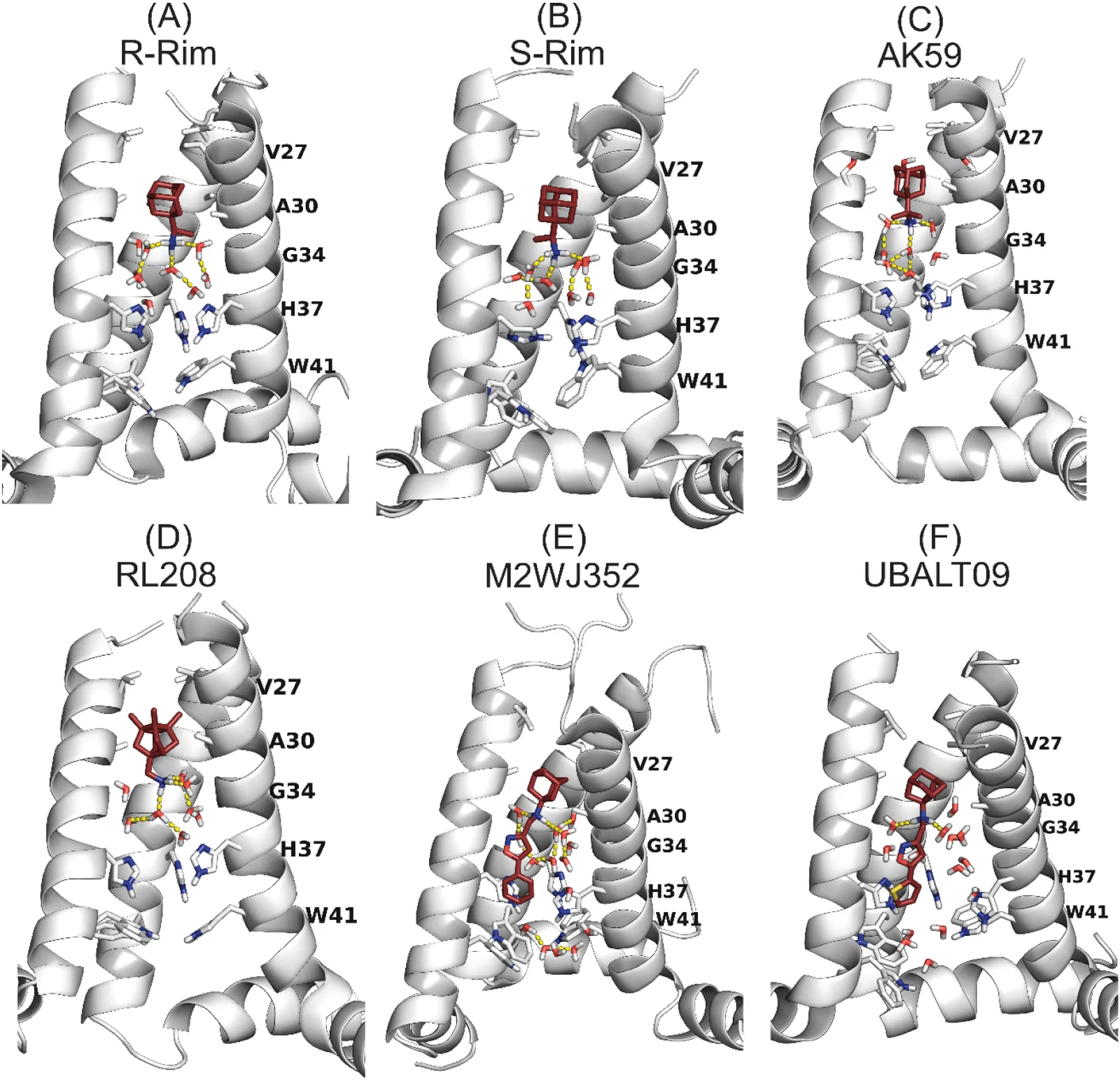
Representative structures from 250 ns-MD simulations of WT M2CD – drug complexes embedded in POPC bilayers using the CHARMM36m force field. (A) R-Rim, (B) S-Rim, (C) AK59, (D) RL208, (E) M2WJ352, (F) UBALT09. The M2 channel is shown with a white cartoon representation, each ligand is shown with a stick representation and colored red and protein residues in gray. Water molecules are shown in stick representation with the oxygen atom in red and hydrogen atoms displayed in white. The fourth helix is transparent for representation purposes.

##### S31N M2CD

Experimental data and MD simulations have shown that, in contrast to WT M2CD, Amt and other adamantyl amine analogs are oppositely oriented in the S31N M2_22–46_ mutant pore with the ammonium group facing the N-terminus (outward) and the adamantyl group inward.^18,71^ We started from docking poses with ammonium group oriented inward, and performed MD simulations of S31N M2CD complexes with R/S-Rmt, AK59, RL208, UBALT09, and M2WJ352. The simulations showed that Rmt, AK59 and RL208 flip from their initial docking poses by 180° (from ammonium-inward to outward, Figure 7, Tables S3, S4). In this orientation, the ammonium group forms hydrogen bonds with Asn31 side chains and backbone carbonyls while the adamantyl group is placed in a broad area defined by Gly34, as previously suggested by MAS NMR data and MD simulations.^18,71,72^ In addition, the drug does not appear to interact with the water molecules between Gly34 or Ala30 and His37 and thus would not be expected to alter the stabilization of hydronium ions. There are no stabilizing van der Waals contacts, and thus water molecules can pass through the channel. The MD simulations of the S31N M2CD complexes with M2WJ352 or UBALT09 led to a conformation of M2CD in which the ammonium group forms hydrogen bonds with the Asn31 amide side chains and waters, and the aryl group is located outward between the Val27 side chains. Although the binding orientation of the S31N inhibitors is confirmed by MD simulations, and UBALT09 restrains the water passage through S31N more efficiently compared to WT, this is only a weak effect (Tables S3, S4, S7).^29^

**Figure 7:**
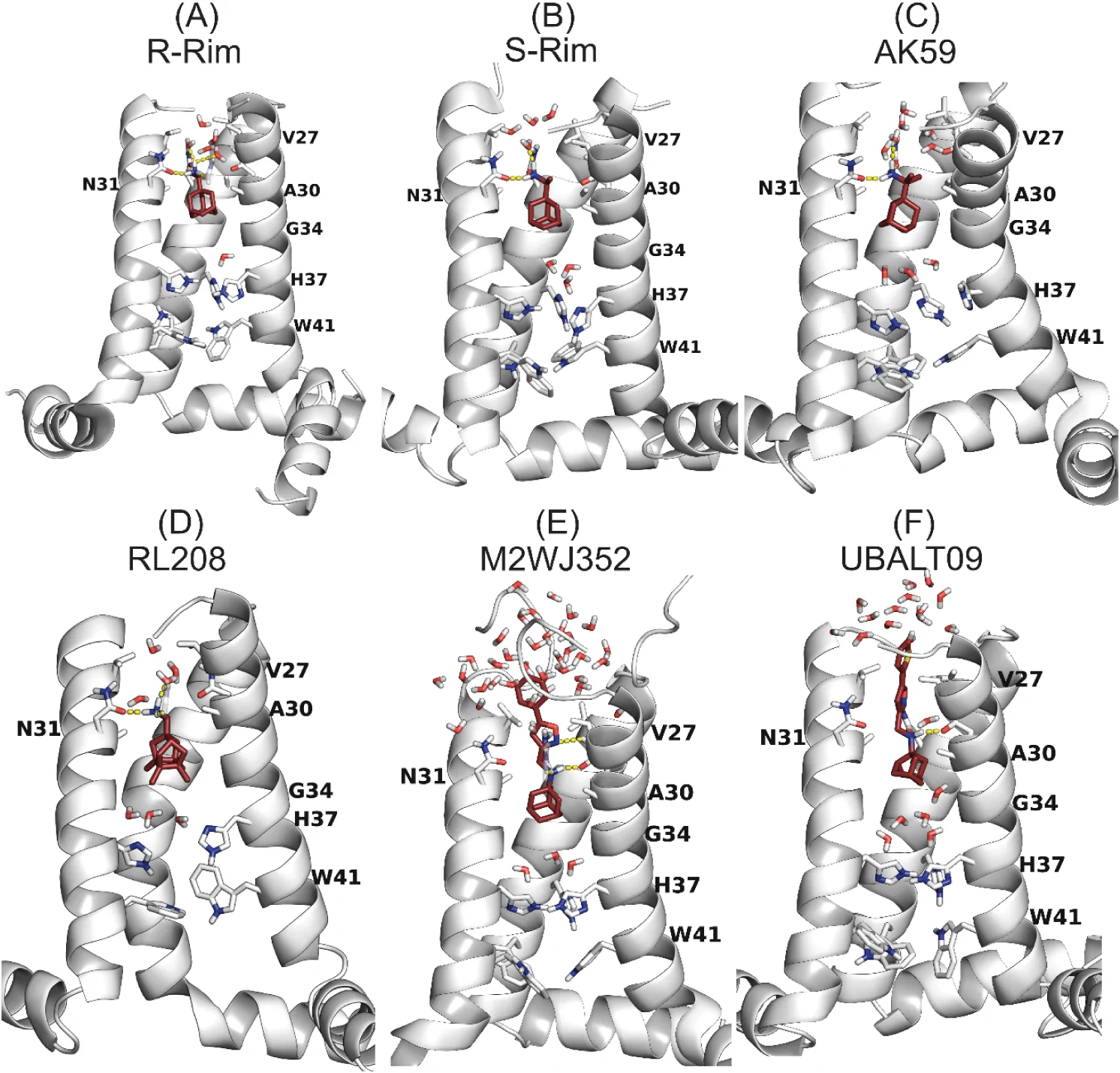
Representative structures from 250 ns-MD simulations of S31N M2CD – drug complexes embedded in POPC bilayers using the CHARMM36m force field. (A) R-Rim, (B) S-Rim, (C) AK59, (D) RL208, (E) M2WJ352, (F) UBALT09. The M2 channel is presented with the cartoon representation in white color, each ligand is colored deep red in stick representation as well as the protein residues in gray color. Water molecules are shown in stick representation with the oxygen atom in red and hydrogen atoms displayed in white. The fourth helix is transparent for representation purposes.

### Liposomal Proton Flux Assay

We used a proteoliposomal functional assay^73^ to examine the ability of the tested drugs to block WT and S31N M2CD and compare with EP and NMR results. In this assay, we used the same WT and S31N M2 constructs containing residues 18 to 60, as in the NMR experiments. The experimental setup of the assay mimics what occurs after the endocytosis of the virus into the infected cell when the M2 channel is activated by the low pH of the endosome^5^ and transports protons into the viral interior. The mechanism of this method relies on a proton gradient that is generated by the addition of hydrochloric acid to a suspension of liposomes that contain M2CD. Proton transport through M2 protein into the liposomes, is monitored by recording the pH of the external solution after the addition of 1 M HCl under continuous fast stirring. A gradual increase in the external pH indicates M2CD-mediated proton conductance. The assay is terminated by the addition of a proton uncoupler, carbonyl cyanide m-chlorophenylhydrazone (CCCP), which results in a rapid recovery of the pH.

Drug inhibition of M2CD activity was qualitatively detected by a decrease or nearly complete loss of pH recovery after the initial HCl addition. The drugs were added to the prepared proteoliposome samples at a final concentration of 50 μM and the system was allowed to equilibrate for 1 h before the first HCl addition. The results for the two protein variants are depicted in Figure 8A–D. In the case of WT M2CD, the results are categorized as strong (Figure 8A), moderate or non- blockers (Figure 8B).

**Figure 8:**
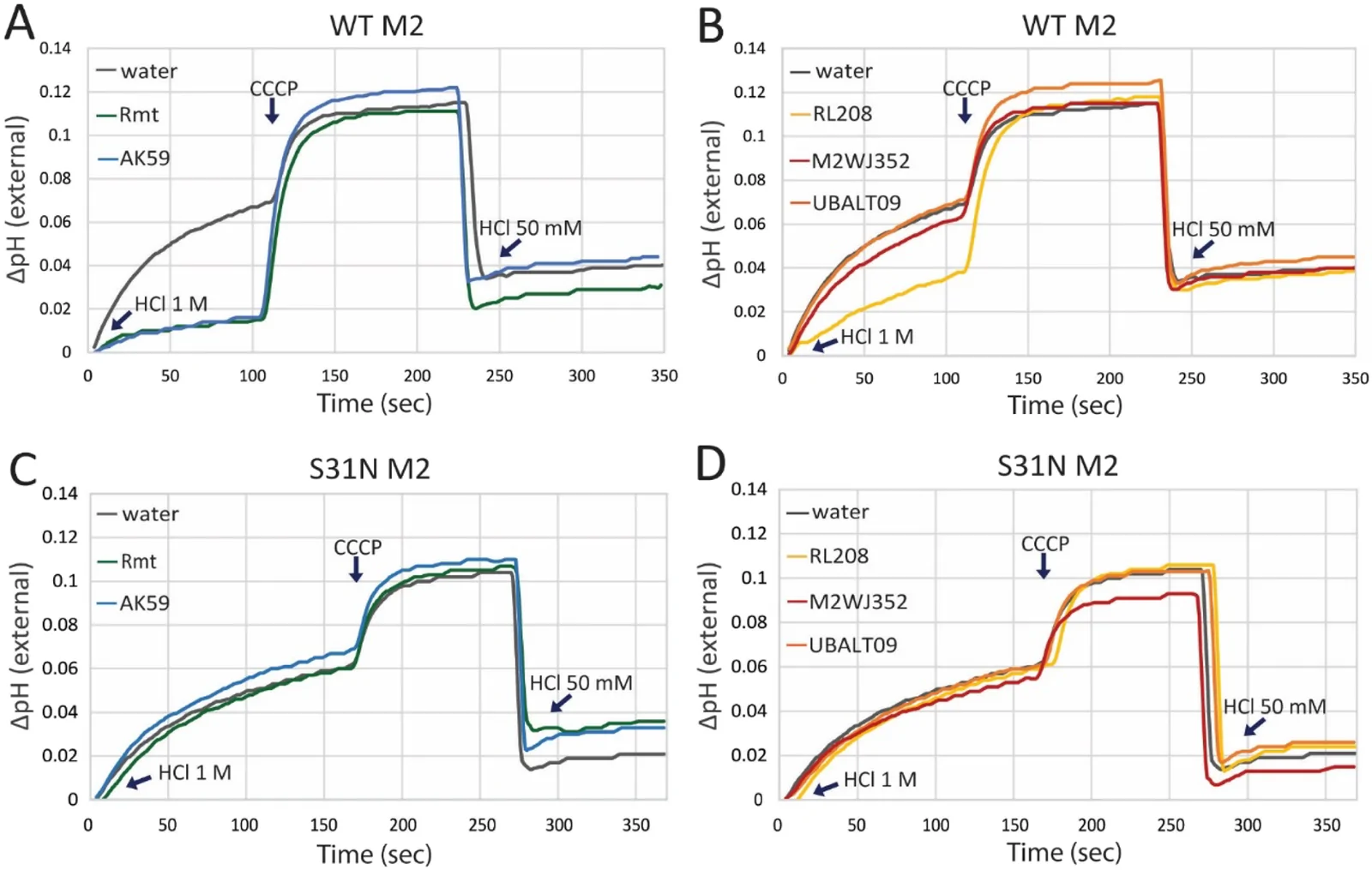
Liposomal proton flux assay applied to WT and S31N M2CD in the presence of five drugs (Rmt, AK59, RL208, M2WJ352, UBALT09). The 5-point moving average method was used to smoothen the data points of the curves. Time zero corresponds to the pH equilibration of the external solution at pH ∼ 6.3 after the addition of 1 M HCl. M2CD activation and proton translocation into the liposome interior are shown as an increase of pH. The addition of the proton uncoupler CCCP terminates the experiment. The buffering capacity of the system is evaluated by adding 50 mM HCl. (A), (B) Proton conduction of WT M2CD is strongly inhibited by 50 μΜ of Rmt and its analog AK59 and partially blocked by RL208. The bulkier ligands M2WJ352 and UBALT09 did not show blocking of the proton channel in 50 μΜ final concentration. (C), (D) Proton conduction of S31N M2CD is not inhibited by any of the drugs tested. The time interval for measuring the proton conduction is extended in this case by 1 min.

Inhibition by a drug, results in a very slow pH recovery after the addition of 1 M HCl. Thus, Amt, Rmt, and AK59 effectively blocked WT M2CD as is shown by the minor pH increase (ΔpH ∼ 0.016) after equilibration of the external solution at pH ∼ 6.3 (Figures 8A, S12). As a control, we added the same volume (50 μL) of the solution (water) used for drug solubilization. The control shows a substantially larger pH increase (ΔpH 0.07), validating the assay. The assay was repeated following the same conditions but using liposomes without M2 protein (Figure S13). There was a slight pH increase (ΔpH < 0.016) after the first addition of HCl, similar to the case of the effective blockers which shows that the liposomes are not perfectly sealed allowing, thus, a small proton leakage. Following the same measurements, M2WJ352 and UBALT09 showed no effective inhibition, as the pH recovery in both cases was almost the same as in the case of the drug-free sample (Figure 8B). RL208 showed a partial blocking of the proton conductance (Figure 8B).

The same procedure was followed for investigating drug inhibition of S31N M2CD. Slower conductance was observed for the S31N channel compared to the WT channel, revealing possibly a tighter tetrameric bundle. Hence, the measurement window was extended by 1 min after the first HCl addition. In all cases, none of the drugs inhibited proton transport into the proteoliposomes (Figure 8 C,D).

This experiment was also performed using the longer construct of WT, M2_21–97_, for the same set of drugs (Figure 9). Interestingly, RL208 blocks proton conductance for this construct to a degree comparable to the full-blockers Rmt, and AK59; a similar, very slow pH recovery is observed after the addition of HCl. In Table S8, the proton conduction rates of M2 of different constructs or variants are reported. The values refer to protons/second/tetramer rates and were calculated as an average flux for the time frame between 25 and 45 s after the first drop in pH. While the smaller molecules, Rmt and AK59, inhibited both M2_18–60_ and M2_21–97_, RL208 demonstrated a notable stronger blocking effect on M2_21–97_.

**Figure 9:**
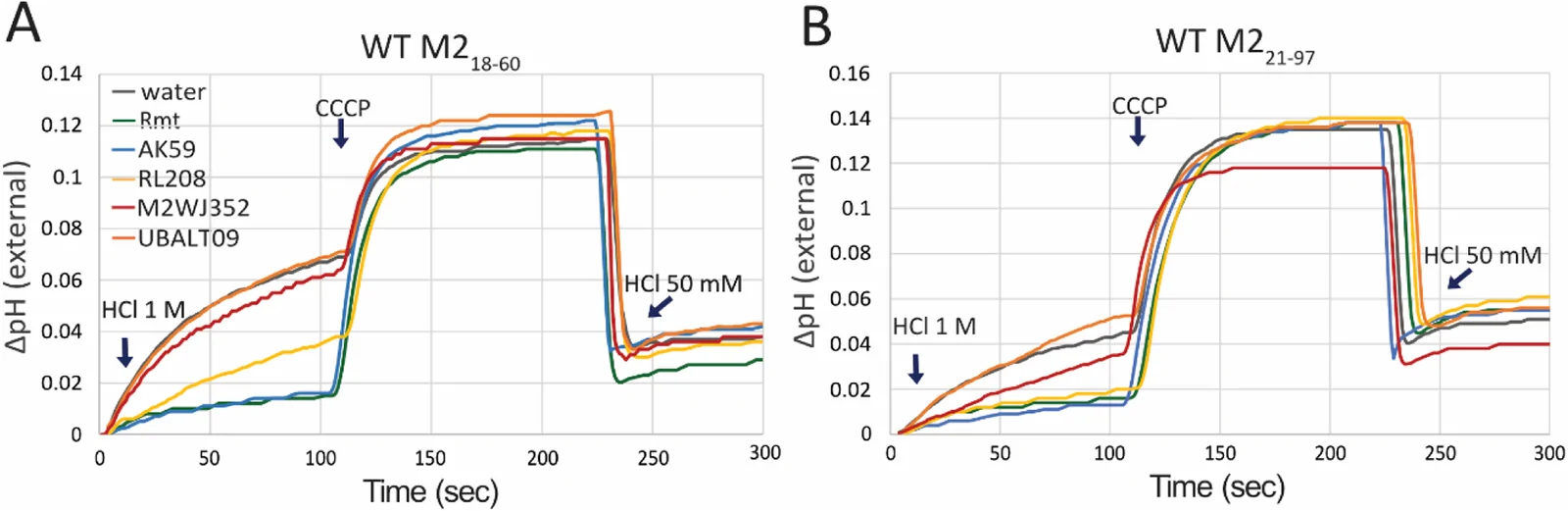
Comparison of liposomal proton flux assay results when applied to a shorter and longer construct of WT M2 in the presence of drugs; (A) M2_18–60_ and (B) M2_21–97_. Note that the concentration of M2_21–97_ is lower than M2_18–60_. For conductance rates per tetramer, see Table S8.

## Discussion

### Drug Binding and Blocking Mechanisms

The tetrameric M2 channel was proposed to transport protons through the breaking and reforming of bonds between His37 residues in adjacent monomers.^14,35^ Indeed, these hydrogen bond interactions have been observed in both CD and FL M2.^49,50,74,75^ Additionally, MAS NMR suggested the presence of hydrogen bonding interactions between His37 and waters using M2_22–46_ ^76^ or M2CD^75^ or M2FL.^14,35,47^ The M2-mediated proton transport is blocked by adamantyl amines and their conjugates by binding to the pore of M2.^17,29,38,44,54,58^

Mechanistically, drug binding and blocking have been proposed to occur a) by drugs occluding the M2 pore and thus preventing the proton transport,^38,43^ b) by acting as a hydronium mimic that interacts with water layers inside the pore that stabilize transient hydronium ions formed during the proton conduction,^44,58^ or c) by indirectly disrupting the imidazole-imidazole hydrogen bond between His37 side chains.^49,50^ It is also possible that multiple mechanistic aspects are needed to explain how different small molecules inhibit the proton transport.

NMR provides a structural view of drug binding, and in particular a readout of changes in the electronic structure and the dynamics of the molecules. We previously saw that it can detect the hydrogen bond between His37 side chains.^49,50^ The His hydrogen-bonding was proposed before^14^ as a mechanism to tune the His37 p*K*_a_’s. Amt, or Rmt bound to WT M2CD disrupts this interaction. Whether the disruption is due to the drug inducing conformational changes of the protein or perturbation of water layers that interact with the histidines has not yet been clarified by NMR, but we are able to see the mechanistic impact they have on the protein at His37. A question that arises next is whether drug binding always includes breaking of His37 H-bonds. This has been detected for the interaction of WT M2 and adamantane-like inhibitors,^50^ but it has not been confirmed for the case of S31N. In previous NMR studies of the second-generation S31N inhibitor, M2WJ352,^57^ it was shown that when the ligand binds, it dehydrates the pore and interrupts the water wire that leads to His37. The evidence came from NMR spectra which indicated that, although M2WJ352 binds far from the His37 with the amino group facing toward the N-terminus, it causes significant suppression of the proton exchange process between His and water. Whether this suggested water wire interruption leads to a disruption of His H-bond has not been so far investigated for S31N M2.

Next, we would like to highlight one more important feature of the M2 structure and that is the allosteric connection between the amphipathic helices and the TM domain, which leads to the importance of using the full-length M2 for future studies. The first evidence of this allosteric connection is the disappearance of the MAS NMR signal of the AH residues when the drug is bound to the pore. On an NMR time scale we are able to detect which part of the protein is dynamic, and in our MAS NMR data the nondetection of the AH means that this part becomes mobile when the drug is bound. A second piece of evidence comes from our previous work^52^ where we measured the spectrum of an N31 M2 variant (Columbia/P0067/2014 H3N2), which differs in the AH residues. The substitution of residues in the AH resulted in loss of the signal from the H-bonded His37, which suggests an allosteric coupling between the two. The scientific community has extensively investigated the mechanistic impact of the M2 inhibitors at the His37. In contrast, we see with the previous examples that finding structural determinants for binding can be more complex when different sequence variants and protein length are being used.

### Blocking and Inhibition of WT M2CD

In order to monitor binding to M2, we recorded multidimensional NMR spectra of WT or S31N M2CD with and without the drug. When we tested the binding of cage alkyl amines Rmt, AK59, RL208 to WT M2CD at a low temperature of 4 °C, we did not observe significant changes in chemical shifts; only small changes (<0.5 ppm) were observed in the ^1^H and ^15^N resonances of His37 side chain in the (H)­NH spectrum. These CSPs suggested nonspecific interactions between the protein and the compound. The small CSPs at 4 °C can be due to the ligand altering the properties of the lipid bilayer^38^ and are consistent with reports that adamantyl amines have slow entrance rates inside the WT M2TM (M2_22–46_) pore by EP studies (Table S1).^17,19^ In addition, MAS NMR data of WT M2CD revealed slow entrance of Rmt^50^ that is consistent with the slow binding of Amt and *k*_on_ measurements of Rmt by EP.^17,39^

Consequently, we increased the temperature of the samples to 55 °C (or 70 °C for S31N) to overcome high kinetic energy barriers for drug entry into the M2CD pore. We previously estimated a high activation energy of ca. 122 ± 16 kJ mol^–1^ for pore binding by tracking binding kinetics using the ^15^N His37 and Gly34 chemical shift changes at different temperatures.^49,50^ The incubation at 55 °C resulted, for Rmt or AK59, in large CSPs in both ^1^H and ^15^N resonances (Figure 1F, G). These large CSPs suggested significant structural rearrangements of the protein,^32,39,49,77^ upon pore-binding as previously reported for Amt in DMPC bilayers^39,77^ and Rmt in DPhPC bilayers.^49,50^ The His37-Nε2 proton resonances at 14.3 and 11.7 ppm, which are characteristic of the imidazole-imidazole hydrogen bonding, were not detected upon Rmt binding and were reduced by 80% in the case of AK59, suggesting that the protein is about 80% in the bound state. This is an example of how subtle differences in ligand’s structure close to the amine group affect binding since AK59 differs from Rmt only by one methyl group attached in the methine linker (CHMe) between the amine and adamantyl group. In contrast, incubation at 55 °C was not sufficient for detecting binding of RL208 to WT M2CD, neither using a pH-shift protocol as an alternative, showing that with bulkier ligands, detecting binding with NMR might be more challenging.

Another observation, that further demonstrates the very slow kinetics of RL208 entering the pore, is that after two years of storage at 4 °C, the NMR fingerprint of the sample with 300 μM concentration of RL208 starts resembling the fully bound form of WT M2CD. In Figure S7, a comparison to the apo and to the Rmt bound M2CD spectrum is shown, and it can be noticed that there are clearly two populations of bound and unbound protein. The results presented here show that a small increase in the size of the drug can affect its entrance and binding to M2CD.

Another interesting point is that RL208 caused partial blocking of proton transport in the liposomal assay when WT M2_18–60_ was incorporated in the lipid membrane. In contrast, in the case of the longer construct (WT M2_21–97_), RL208 behaved as a full blocker of the channel. That could be attributed to different dynamics of the longer protein that can help to accommodate a bulky ligand into its pore. As regards the slow drug binding kinetics of RL208 in the NMR sample compared to the fast binding kinetics in the liposomal assay samples, we cannot rule out the contribution of the different membrane composition.^50^ The liposomal assay samples have a much higher lipid to protein ratio (400:1 by mass compared to 1:1 by mass used in NMR) and use an *E. coli* lipid extract rather than single component lipids. Changes in lipid properties, such as the hydrophobic thickness or pH, or the presence of a drug, were shown to induce substantial changes in the conformation of the transmembrane helix of M2 for both the TM peptide and M2CD with a spin label substituted at R45.^78–80^ By contrast, the M2CD preparations used in this and previous work,^50^ were shown to consistently assemble in the dimer-of-dimers state in single component lipids as well as lipid conditions that included cholesterol and in a complex mixture of lipids that mimics the viral membrane.^50^

In the case of the bulkier molecules, M2WJ352 and UBALT09, which are not blockers of WT, we did not observe clear binding or blocking of WT M2 channel, as expected. However, UBALT09 showed partial binding in NMR at a high drug concentration. Although the presence of both bound and unbound M2 are evident in the (H)­CANH spectrum, the affinity may be rather low in this assay, since a 20-fold higher drug concentration was needed. Additionally, we have previously reported in MD simulations and binding free energy calculations studies,^27^ that aryl conjugates of amantadine can bind weakly to WT M2TM, but without blocking the channel in EP. Consequently, we can derive from these results that UBALT09 may bind weakly to WT M2CD but does not block the channel as shown in our liposomal assay results. This example also shows that, in order to investigate the M2 druggability, the use of only one biophysical method to evaluate the drug’s mechanism of action is not always sufficient. It is of note, that limitations coming from the experimental approach can sometimes hinder inhibition. For example, a TEVC assay that uses M2FL expressed in oocytes, detects inhibition of the AM2 proton current during a 2 or 5 min measurement and this is the time window that the channel gets exposed to the drug. If a drug has very slow *k*_on_, the assay may not detect a true inhibitor that shows potency in antiviral assays. One example is that of a Rmt analog in ref 19, where only 27% blocking was observed at 2 min against WT M2 in the TEVC assay, but low micromolar EC_50_ was observed in an antiviral assay. On the other hand, if the *k*_off_ is too fast but *k*_on_ is also fast, then the drug might show proton current blocking in TEVC but might not be able to inhibit the virus in vitro, considering that relatively high concentrations are applied in TEVC assays. Similar discrepancies have been previously mentioned.^21,27^

### Blocking and Inhibition of S31N M2CD

The Amt—aryl conjugate molecules, such as M2WJ352 and UBALT09, were developed in order to block S31N M2 because of the ineffectiveness of previous adamantyl amines against the S31N strains. Discrepancies were observed between electrophysiology results and NMR or liposomal assay data. Although the results from NMR and the liposomal functional assay are in good agreement, showing that these two molecules are not blocking S31N M2CD, electrophysiology categorizes them as strong inhibitors of the S31N channel.^29^ In this case, the discrepancy might come from the use of M2CD for the NMR and liposomal assay, while M2FL was used for electrophysiology. On the other hand, we used S31N M2CD for the MD simulations, and it showed that the two ligands orient themselves with their substituted amine group facing toward the Asn31 (Figure 7). This orientation captured by the MD simulations agrees with what has been previously reported from NMR measurements of the S31N M2TM in complex with those selective inhibitors.^57^ That could explain the reason why we do not observe the H-bond disruption of the His37 in NMR, if the amine group interacts with the water clathrate without the disruption of His37 interactions. In that case, there might also be no substantial helical rearrangement, which is distinct from drug-bound WT M2CD, in which case NMR experiments detected helix rearrangement, including allosteric effects in the amphipathic helix residues.^32,50^ Nevertheless, the simulations did not show an effective blockage of the water passage which is consistent with the rest of the methods used to monitor drug activity (Table S7).

Additionally, the solution and NMR data of S31N M2TM – M2WJ352 complex, mentioned before, in DPC micelles^29,56^ and DMPC or a virus mimetic membrane,^57^ showed that Asn31 has the largest CSPs when the drug is bound to the M2 pore followed by His37 and Gly34. In our NMR experiments with S31N M2CD, we did not observe significant CSPs at Asn31 for either drug tested. It is worth noting that in previous studies on S31N M2 and inhibitors, S31N M2_19–49_ or S31N M2_22–46_ were used, in which the amphipathic helices are missing.^29,56,57^ It seems that AHs could generate a structural barrier for those drugs to enter the S31N M2CD pore i.e., induce conformational changes that hinder drug binding to the M2 tetramer.

Altogether, we could not so far detect blocking or binding of selective inhibitors to S31N M2. A future use of M2_21–97_ or M2FL in that case is essential to rule out the possibility of construct length being the factor for activity discrepancies with previous reports. However, we cannot either rule out that different inhibition mechanisms could be present, as those inhibitors show activity in plaque reduction assays. It has been recently reported that some amantadine analogues could act on multiple or even complementary antiviral targets in S31N viruses and act at different stages of the virus life cycle, such as the virus cellular entry.^81^

## Conclusions

In this work, we combined complementary biophysical approaches to further explore our previous NMR findings and to investigate the binding and blockage of WT or S31N M2 by cage alkyl amine containing inhibitors. We applied MAS NMR complemented with MD simulations to assess the potential blocking mechanism for both variants, while a liposomal proton flux assay was performed along with EP measurements to assess the ability of the compounds to inhibit the M2-mediated proton conductance. In addition, we developed two MAS NMR sample-preparation protocols that helped overcome the high kinetic energy barriers associated with drug entry into the M2CD pore.

We demonstrated that low pH allows more reliable equilibration with drugs, presumably by opening of the tetramer at the C-terminal side of the membrane upon His37 imidazole protonation, after which NMR data can be subsequently recorded at alkaline pH in the closed-state conformation.^43–46,53,82^ We further found that even small increases in drug size can dramatically affect the kinetics of binding to the M2CD pore, in contrast to M2TM, emphasizing the conformational constraints imposed by the AHs.^39,49^ Disruption of the imidazole-imidazole hydrogen bonding was a clear indicator for compounds that block WT M2-mediated conductance. On the other hand, a lack of disruption was observed for S31N M2CD, even for drugs previously reported to be active in EP. This discrepancy does not appear to be an effect caused by the high protein concentration used in the NMR samples, as the more dilute conditions of the liposomal assay similarly showed no evidence of blocking. Herein, our study focused primarily on protein-drug interaction using the 18–60 conductance domain of WT and S31N variants. In the process, the importance of using a structure that is closer to the native structure of M2 protein has become clear. In other words, it will be important to compare these results with testing of full length M2 in the future and using NMR methods that include isotopically labeled drugs. Such studies may provide deeper understanding of the drug inhibition mechanism of the channel’s proton conductance and may subsequently facilitate the rational design of new M2 inhibitors.

## Experimental Section

### Magic Angle Spinning Solid State NMR Spectroscopy

The M2 conductance domain (M2CD; residues 18 to 60) of influenza A was obtained and purified following methods described previously.^12,32,51,55^ Briefly, the M2 amino acid sequence from the A/Udorn/1972 H3N2 strain, designated as wild type (WT), was utilized, incorporating C19S and C50S mutations. The S31N variant was generated from the WT sequence by introducing the corresponding mutation. ^15^N and ^13^C labeled M2 was expressed using U–^13^C-glucose, and ^15^N-ammonium chloride as the sole carbon and nitrogen sources, respectively. *Escherichia coli* was used to express the protein in inclusion bodies, which were then resuspended in 6 M guanidinium and subsequently purified using a nickel column. Cyanogen bromide was used to cleave the His-9 and TrpLE tags. Protein was then subjected to C4 HPLC column purification following a gradient elution using a mixture of 3:2 isopropanol:acetonitrile. The HPLC fractions were dialyzed against water and then lyophilized. The resulting lyophilized protein was resuspended in NMR buffer (40 mM sodium phosphate, 30 mM glutamate, 3 mM sodium azide, pH 7.8) containing octyl glucoside detergent and reconstituted in deuterated DPhPC lipids (FB Reagents) with a lipid:tetramer molar ratio of 25:1 or 1:1 by mass. Finally, the samples were packed in a 1.3 mm rotor at room temperature using a benchtop centrifuge and had an estimated tetramer concentration of 12 mM. For the drug binding experiments, a solution of 300 μΜ or 6 mM of drug was equilibrated with the membrane pellet from one rotor (2 to 3 μL) in a volume of 200 μL. This is sufficient total drug, since the estimated drug:tetramer molar ratio is 2.5:1 or 50:1 during incubation. The final concentration in the membrane pellet depends on the partitioning between the aqueous phase and the pellet. For the experiments where we changed temperature, the packing of the rotor with the sample was done at ambient temperature (∼21 °C). After the first measurement the rotor was kept for 2–5 h (unless otherwise indicated) in a 55 °C water bath. Samples were stored at 4 °C and the temperature during NMR measurements was ∼25 °C. For the drug binding measurements by changing the pH, the drugs were added by pushing the membrane pellet out of the rotor into a 300 μM (unless otherwise indicated) drug solution in citrate buffer of the desired pH (40 mM disodium phosphate, 22 mM citric acid, 3 mM sodium azide). Following overnight incubation at 4 °C, the pellet was moved to the same buffer, but at pH 7.8 for measurement.

All spectra were acquired on an 800 MHz Bruker Avance III spectrometer using a Bruker 1.3 mm HCN probe. The spinning frequency was 55 kHz and the gas flow was set to 900 L per hour, the temperature of cooling gas was set to 243 K, which results in an estimated sample temperature of 25 °C based on the chemical shift of water. The proton spectrum was referenced by setting the chemical shift of water to 4.7 ppm. 2D (H)­NH spectra, were processed with a cosine squared function using 8 ms in the proton dimension, and 10 or 26 ms in the nitrogen dimension. Proton and ^15^N hard pulse powers were 100 kHz and 80 kHz respectively. For ^1^H to ^15^N and ^15^N to ^1^H CP, the contact time was 800 and 550 μs, respectively, with a 20% linear ramp on ^1^H and constant irradiation of ^15^N at 38.5 kHz. 3D (H)­CANH spectra were processed with a cosine squared function using 10 ms in the proton dimension, 15 ms in the nitrogen dimension, and 9 ms in the carbon dimension. Proton, ^15^N and ^13^C hard pulse powers were 100 kHz, 80 kHz, and 80 kHz, respectively. We set the H–Cα CP contact time to 600 μs with a 20% linear ramp on ^1^H centered at about 95 kHz and 41.2 kHz on ^13^C, a Cα-N CP with a contact time of 10 ms using 43.0 kHz and 19.6 kHz on ^15^N and ^13^C, respectively, and a 30% tangential ramp on the ^15^N channel. The N–H CP had a contact time of 400 μs, a 20% linear ramp on ^1^H centered at about 93 kHz and 38.5 kHz on ^15^N. All spectra were acquired with frequency swept TPPM^83^ ^1^H decoupling at 12 kHz during indirect acquisition times and WALTZ-16 ^84^ decoupling at 10 kHz on both ^13^C and ^15^N channels. For water suppression, the MISSISSIPPI^85^ method was used at 13 kHz. Spectra were processed in Topspin 3.5 (Bruker) and analyzed in Sparky.^86^

### Computational Biochemistry

#### Molecular Docking Calculations

Protein and ligand preparation for the docking calculations are included in the Supporting Information. The WT M2_22–62_ structure with PDB ID 2L0J^35^ from NMR in phospholipid bilayers was used to prepare the M2_18–60_ tetrameric structure. Mutant S31N M2(18–60) protein complexes were obtained by manually mutating serine 31 to asparagine in the PDB ID 2L0J^35^ protein. M2_18–60_ was superimposed to complex WT M2_22–46_ - Amt, with PDB ID 2KQT,^38^ to introduce ligand coordinates. Protein WT M2_18–60_ and ligand Amt and its coordinates were used as reference coordinates for the docking calculations. Amt, R-Rim, S-Rim, AK59, RL208, and M2WJ352, UBALT09, were docked to the WT M2_18–60_ protein, with GOLD 5.2 software (GOLD Suite, version 5.2; Cambridge Crystallogr. Data Cent. Cambridge, U.K., 2015) and the Gold Score.^87^ For the docking calculations of the compounds, the water clathrate between the ligands and His37 tetrad was maintained as part of the protein structure in agreement with the X-ray structures.^44^ All the protein atoms within 10 Å of the reference ligand were used for binding site definition. For the docking calculations, 30 genetic algorithm runs were applied (instead of 10 runs as the default choice), and the “allow early termination” option (which is also not the default choice) was chosen while all other options were kept as default. The resulting docking poses were visually inspected using the UCSF Chimera package.^88^ Docking calculations in M2 constructs were also performed with Glide (Glide Schrödinger LLC, New York, NY, 2012. Glide, version 5.7; Glide Schrödinger LLC, New York, NY, 2011) using the scoring function Glide XP,^89^ an empirical function that approximates ligand binding free energies, as well as the induced-fit method. As a measure of docking calculation accuracy, the experimentally determined coordinates of Amt in PDB ID 2KQT^38^ were compared with the docking results. The root-mean-square deviation (RMSD) of the docked Amt compared to the reference position, with either GOLD 5.2 or Glide, was <0.1 Å, which suggested that docking calculations would be accurate also for the other docked ligands. The generated complexes of M2_18–60_ were used as starting structures for MD simulations.

#### MD Simulations

The complexes between R-Rim, S-Rim, AK59, RL208, and M2WJ352, UBALT09 and M2CD from docking calculations were aligned with the membrane using the Orientations of Proteins in Membranes (OPM) Server.^90^ The insertion of the OPM – protein complexes into the bilayer was performed with the Desmond System Builder utility of Maestro (Schrödinger Release 2021–2: Desmond Molecular Dynamics System, D. E. Shaw Research, New York, NY, 2021. Maestro-Desmond Interoperability Tools, Schrödinger, New York, NY, 2021–2). Protein–ligand complexes were inserted in pre-equilibrated hydrated bilayers of POPC lipids expanding from the furthest vertex of the protein to the simulation box edge 13 Å in the *x,y*-plane and 17 Å in the *z*-axis. The simulation box dimensions were (75 × 75 × 100 Å^3^) and periodic boundary conditions were applied. For the M2CD systems, the system contained 130 – 140 lipids per tetramer. Sodium and chloride ions were randomly added in the aqueous phase to neutralize the system based on a Monte Carlo approach, as implemented in Desmond. The NaCl concentration was chosen to be the physiological 150 mM. The CHARMM36m force field^69,70,91,92^ was used for protein, lipid, and ions and the intermolecular interactions, and TIP3P for waters.^93^ Ligands were parametrized with SwissParam^94^ for the MD simulations. In the SwissParam force field, the dihedral angle terms, the harmonic part of the bonds, angles and improper terms, and charges were taken from the MMFF94 ^95^ force field and van der Waals parameters from the closest atom type in the CHARMM22 ^96^ force field. For the MD simulations with the CHARMM36m force field, the CHARMM36 implementation of TIP3P waters was used. To assign CHARMM parameters, the Schrödinger VIPARR utility (Schrödinger Release 2021–2: Desmond Molecular Dynamics System, D. E. Shaw Research, New York, NY, 2021; Maestro-Desmond Interoperability Tools, Schrödinger, New York, NY, 2021) was used. All simulations were performed with the Desmond MD simulation algorithm^97^ (Schrödinger Release 2021–2: Desmond Molecular Dynamics System, D.E. Shaw Research, New York, NY, 2021. Maestro-Desmond Interoperability Tools, Schrödinger, New York, NY, 2021). The particle mesh Ewald method was used for the calculation of long-range electrostatic interactions^98^ with a grid spacing of 0.8 Å. The Shake^99^ method was used to keep all bonds with hydrogen rigid, at ideal lengths and angles. The Nose – Hoover thermostat^100^ was utilized, and the Martyna – Tobias – Klein method^101^ was used for pressure control. The equations of motion were integrated using the multistep RESPA integrator^102^ with an inner time step of 2.0 fs for bonded interactions and nonbonded interactions within the cutoff. An outer time step of 6.0 fs was used for nonbonded interactions beyond the cutoff. Periodic boundary conditions were applied. For all MD simulations, the same relaxation protocol was used and run on a CPU. In short, two rounds of steepest descent minimization were performed: one with a maximum of 2000 steps and a harmonic restraint of 50 kcal mol^–1^ Å^–2^ on all heavy solute atoms and a second of 10000 steps without restraints until convergence to 1 kcal mol^–1^ Å^–1^. Next, a series of MD simulations were performed to smoothly relax the system; 200 ps starting at 10 K with gradual heating until 310 K in the NVT ensemble with solute heavy atoms restrained with a force constant of 50 kcal mol^–1^ Å^–2^. A temperature of 310 K was used to ensure that the membrane state was above the melting temperature for POPC lipids.^103^ The heating was followed by equilibration runs. First, a 1 ns simulation under NPT conditions was conducted, with solute heavy atoms restrained with a force constant of 10 kcal mol^–1^ Å^–2^ to equilibrate solvents and lipids. This was followed by two 1 ns MD simulations with the NPT ensemble. Briefly, harmonic constraints were gradually decreased from 10 to 2 kcal mol^–1^ Å^–2^ on solute heavy atoms while 2 kcal mol^–1^ Å^–2^ constraints were retained on all other solute atoms. Next, the harmonic constraints were removed except for a 2 kcal mol^–1^ Å^–2^ set on protein C_α_ atoms. Finally, a 5 ns unrestrained MD simulation in NPT conditions was performed before the production phase. This equilibration protocol was followed by 250 ns unrestrained MD simulations in the NPT ensemble with CHARMM36m force field. Within this simulation time, the RMSD of the protein backbone C_α_ atoms of the TM domain (RMSD­(Cα)) reached a plateau, and the system was considered equilibrated and suitable for statistical analysis. The coordinates of the systems were saved every 100 ps. MD simulations were performed in workstations with GPUs using the GPU implementation algorithm of the MD simulation code provided by Desmond software, (Schrödinger Release 2021–1: Desmond Molecular Dynamics System, D. E. Shaw Research, New York, NY, 2021; Maestro-Desmond Interoperability Tools, Schrödinger, New York, NY, 2021).

#### Liposomal Proton Flux Assay

This assay was performed as described previously.^73^ Briefly, M2CD proton channels were reconstituted in a glass tube into liposomes by mixing 25 μg of protein, 750 μL of *Escherichia coli* polar lipid extract (Avanti Polar Lipids) in chloroform (∼10 mg of lipids), 5 μL of 0.04 mM valinomycin solution in ethanol and 350 μL of methanol for homogenized mixing that provided a solution. The solvents were evaporated under continuous nitrogen gas and a thin film was obtained. The films were redissolved in 750 μL of chloroform and dried down again under a nitrogen stream in order to form high quality transparent lipid films and were lyophilized overnight to remove any solvent trace. The dried films were then resuspended in strongly buffered internal liposome buffer (IVB; 26 mM potassium citrate, 17 mM citric acid, 28 mM sodium citrate, 25 mM K_2_HPO_4_, 25 mM Na_2_HPO_4_, 6 mM NaN_3_; pH 7.7) to form liposomes which were then extruded 11 times through 0.2 μm polycarbonate membrane to get a homogenized size distribution. Buffer was exchanged on a PD-10 column (GE Health Sciences) such that the external liposome buffer was a weak buffer (EVB; 4% v/v IVB, 117 mM KCl, 117 mM NaCl, 6 mM NaN_3_, pH 7.7). Every tested sample contained 2.25 mL of liposome mixture which consists of 5 mg/mL lipids, 0.6 μΜ M2 tetramer, and 0.1 μΜ valinomycin as a potassium ionophore that transports potassium ions in the opposite direction of protons. The initial decrease of external pH was initiated by the addition of 3.75 μL of 1 M HCl under continuous fast stirring. The experiment was terminated by the addition of 3 μL of the proton uncoupler carbonyl cyanide m-chlorophenylhydrazone (CCCP). The buffering capacity of the final mixture was evaluated by the addition of 7.5 μL of 50 mM HCl. M2 drug inhibition was investigated by adding 22.5 μL of 5 mM stocks in water (or ethanol in the case of M2WJ352) for a 50 μΜ concentration in the final tested samples. The drugs were added 1 h before the first HCl addition. The addition of 22.5 μL of water or ethanol was tested as separate experiment controls and no change in M2 proton conduction was observed.

## Supplementary Material



## Notes

Tables S1–S8, Figures S1–S13, additional experimental details and complementary results from NMR, liposomal proton flux assay experiments, and MD simulations (PDF)
