# Supplementary material for: Binding and Blocking of Influenza A WT and S31N M2 by Cage Alkyl Amine-Containing Inhibitors
Source: ACS Omega. 2026 Oct 23;11(43):66755–71. doi: 10.1021/acsomega.6c06359 (PMC13737014; doi:10.1021/acsomega.6c06359)
Supplement: Supplementary file 1 [file ao6c06359_si_001.pdf]

SUPPORTING INFORMATION

Binding and Blocking of Influenza A WT and S31N M2 by Cage Alkyl Amine Containing Inhibitors

Marianna Stampolaki, [1,2] Kumar Tekwani Movellan, [1,3] Kyriakos Georgiou, [2] Abel Cherian Varkey, [1] Andreea L. Turcu, [4,5] Santiago Vázquez, [4,5] Stefan Becker, [1] Antonios Kolocouris, [2*] Loren B. Andreas [1*]

[1] Department of NMR-Based Structural Biology, Max Planck Institute for Multidisciplinary Sciences, Am Fassberg 11 Göttingen, Germany

[2] Laboratory of Medicinal Chemistry, Section of Pharmaceutical Chemistry, Department of Pharmacy, National and Kapodistrian University of Athens, Panepistimiopolis-Zografou 15771, Greece

[3] Department of Chemistry and Biochemistry, University of Delaware, Newark, Delaware 19716, United States

[4] Laboratori de Química Farmacèutica, Facultat de Farmàcia i Ciències de l'Alimentació, Universitat de Barcelona, 08028 Barcelona, Spain

[5] Institute of Biomedicine of the University of Barcelona, IBUB, 08028 Barcelona, Spain

Keywords: adamantyl amine; influenza A; S31N M2; liposomal proton flux assay; magic angle spinning; MD simulations; solid state NMR

Previous electrophysiology results

Table S1. Percent reduction of full-length A/Udorn/72 M2 WT or S31N current.

Compound	WT M2	S31N M2	Kd[b]	K_{on}	K_{off}
Rmt [1, b]	96 ± 1[a]	1 ± 0.2	7 μM	416 M^{-1} s^{-1}	0.003 s^{-1}
AK59 [1]	90 ± 0.2	21 ± 2	13 μM	230 M^{-1} s^{-1}	0.003 s^{-1}
RL208 [2]	84.2 ± 0.5	2.0 ± 1.0			
M2WJ352[3, b]	20	91			
UBALT09 [4, b]	7.1 ± 1.8	78.6 ± 1.8			

[a]Mean ± SEM calculated at 2 min of drug (100 μM) perfusion (N=3); [b]values taken from the indicated references.
[b] at 100 μM drug concentration tested against WT M2.

MAS NMR Spectrometry

Table S2. Effects of compounds Rmt, AK59, RL208, M2WJ352, and UBALT09 on two influenza A M2CD variants in two conditions using drug:tetramer molar ratio 2.5:1.

Compound	CSPs in ^{15}N of G34 and side chain His37 / His37-His37 hydrogen bond			
	Drug binding at elevated temperature		Drug binding with pH shift	
	WT	S31N	WT	S31N
Apo	- / yes	- / yes	- / yes	- / yes
Rmt [5]	LS[a] / no	NB / yes	LS / no	NB / yes
RL208	NB / yes	NB / yes	LS / partial	NB / yes
AK59	LS / yes (20%)	NB / yes	LS / no	NB / yes
M2WJ352	NT	NT	NB / yes	NB / yes
UBALT09	NT	NB / yes	LS / partial [b]	NB / yes

[a] LS = large shifts, NB = no binding, NT = not tested; [b] drug:tetramer ratio was 50:1 by mol; The threshold for LS is at least one CSP > 0.2 ppm.

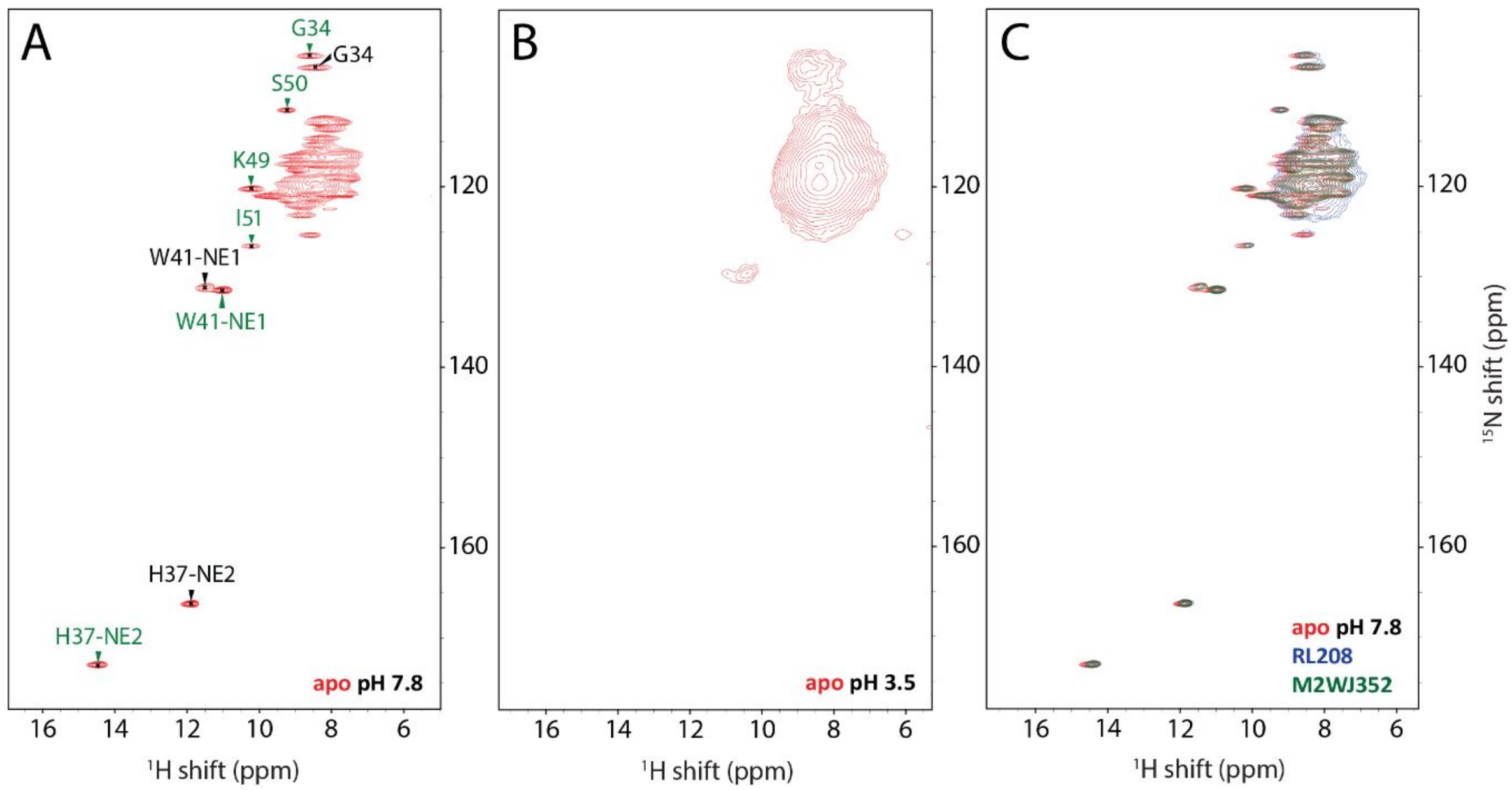

Figure S1. (H)NH spectra of WT M2CD (A) at pH 7.8, (B) at pH 3.5, (C) without (red) and in the presence of RL208 (blue) or M2WJ352 (green) at a drug:tetramer molar ratio of 2.5:1 inside the incubation solution of 300 μM drug concentration. The samples were reconstituted in DPhPC bilayers at pH 7.8 using a lipid/tetramer molar ratio of 25:1. Spectra with drug were recorded at pH 7.8 after relaxing the system at acidic pH 3.5 in the presence of the drug and switching back to alkaline pH. All spectra were recorded on an 800 MHz Bruker spectrometer with a 3-channel 1.3 mm probe at 55 kHz MAS. Residue labels of the dimer-of-dimers structure are colored black for chain A and green for chain B.

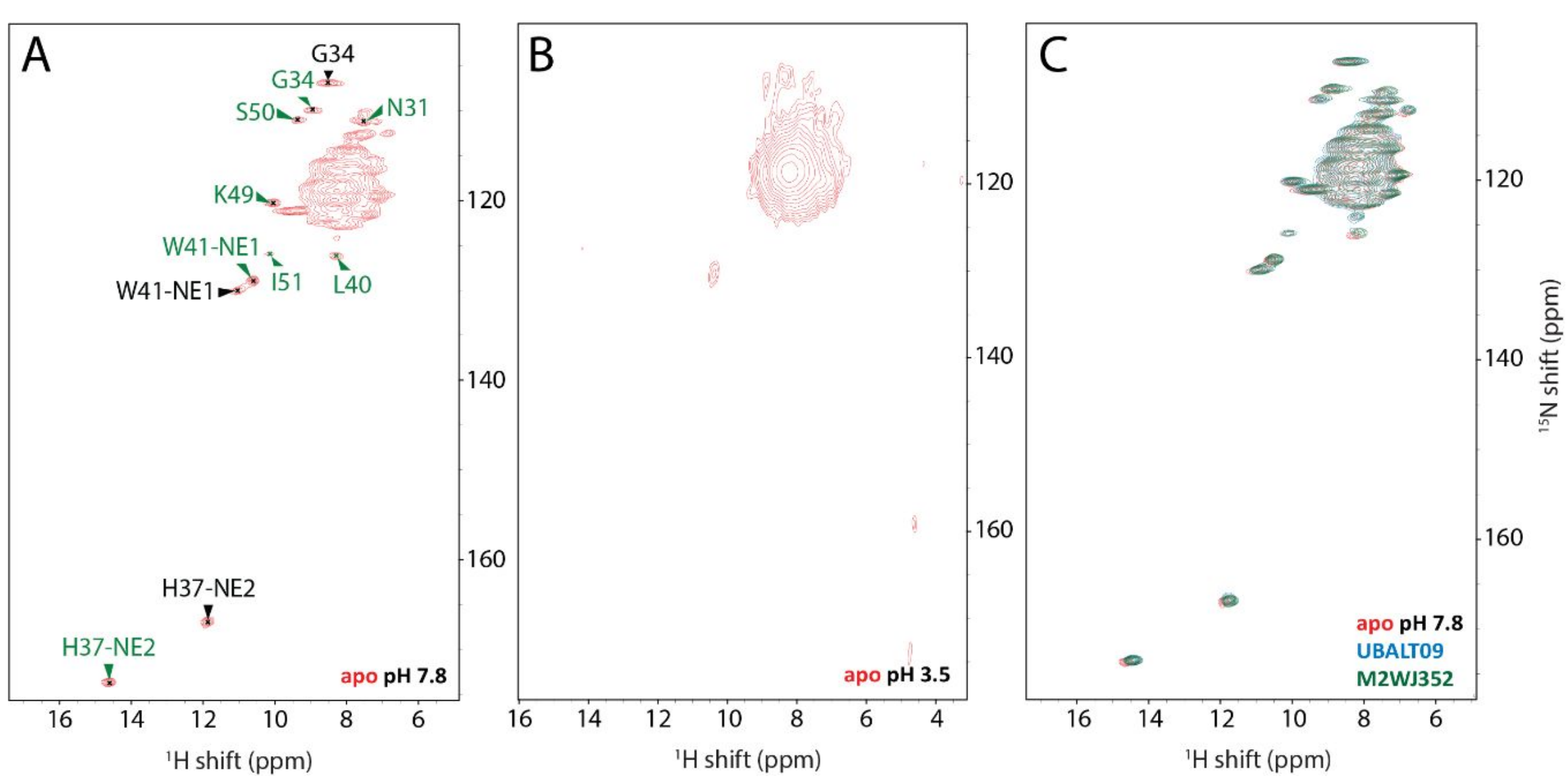

Figure S2. (H)NH spectra of S31N M2CD, (A) at pH 7.8, (B) at pH 3.5, (C) without (red) and in the presence of UBALT09 (blue) or M2WJ352 (green) at drug:tetramer ratio 2.5:1 by mol

(300 µM drug conc.). The samples were reconstituted in DPhPC bilayers at pH 7.8 using a lipid/tetramer molar ratio of 25:1. Spectra with drug were recorded at pH 7.8 after relaxing the system at acidic pH 3.5 in the presence of the drug and switching back to alkaline pH. All spectra were recorded on an 800 MHz Bruker spectrometer with a 3-channel 1.3 mm probe at 55 kHz MAS. Residue labels of the dimer-of-dimers structure are colored black for chain A and green for chain B.

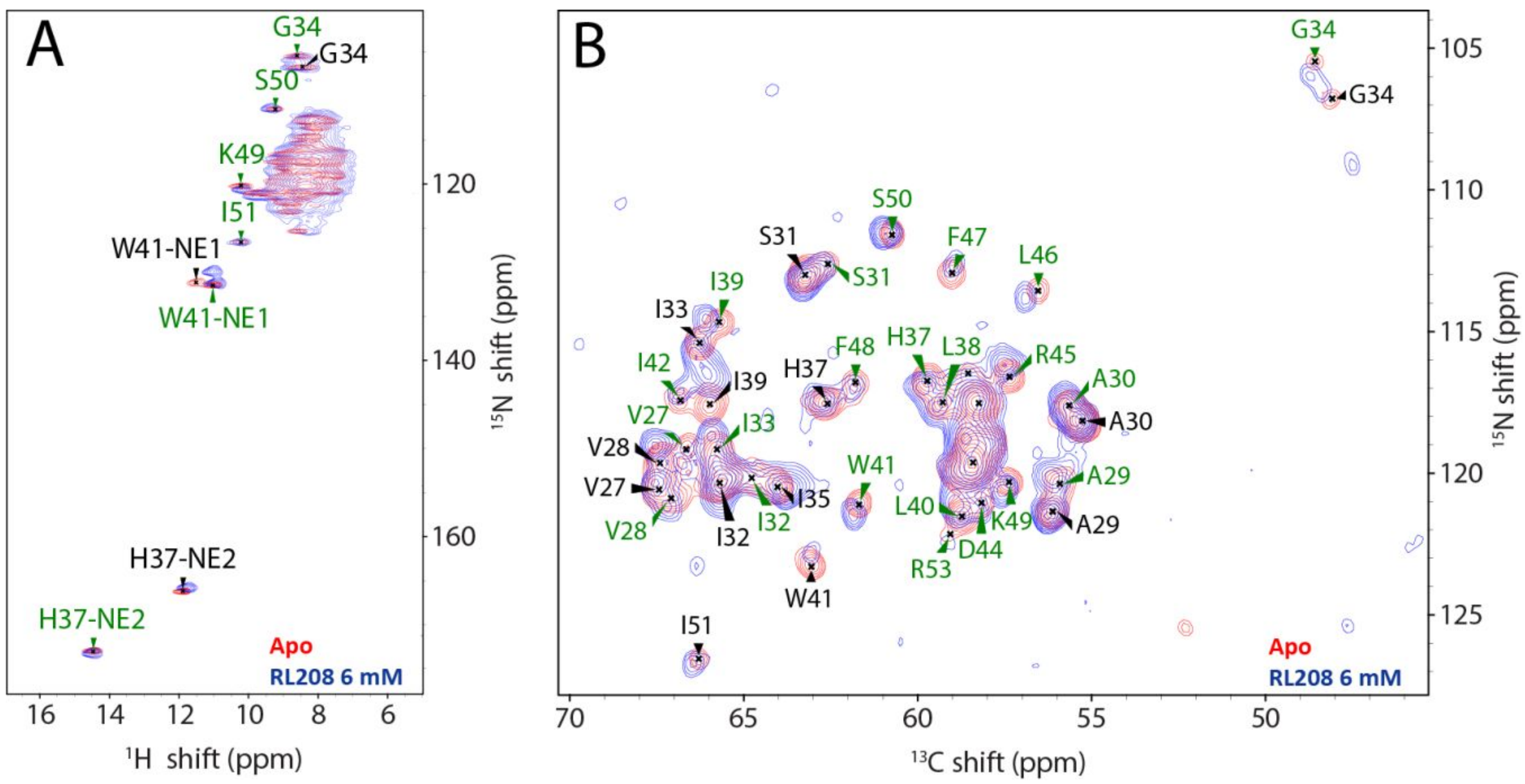

Figure S3. Monitoring the ^{1}H, ^{15}N, and ^{13}C chemical shift perturbation in WT M2CD resonances in the presence of RL208 at a drug:tetramer molar ratio of 50:1 inside the incubation solution of 6 mM drug concentration. (A) (H)NH spectrum of WT M2 with (blue) and without RL208 (red). (B) ^{15}N-$^{13}C\alpha$ projection of the (H)CαNH spectrum of WT M2 with (blue) and without RL208 (red). The samples were reconstituted in DPhPC bilayers at pH 7.8 using a lipid:tetramer molar ratio of 25:1. Both spectra were recorded at pH 7.8 after relaxing the system at acidic pH 3.5 in the presence of the drug and switching back to alkaline pH. All spectra were recorded on an 800 MHz Bruker spectrometer with a 3-channel 1.3 mm probe at 55 kHz MAS. Residue labels of the dimer-of-dimers structure are colored black for chain A and green for chain B.

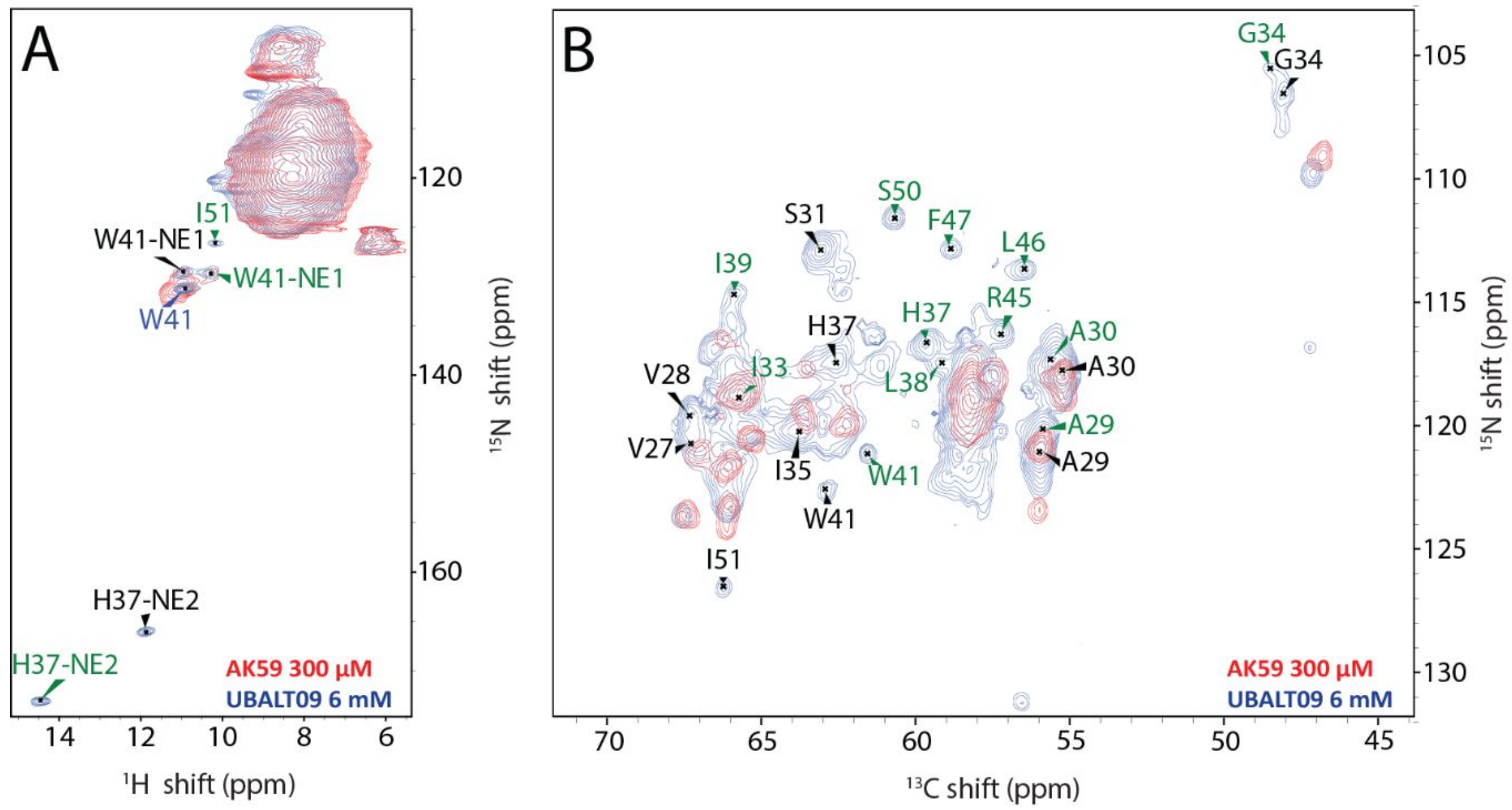

Figure S4. Monitoring the ^1H and ^{15}N chemical shift perturbation in WT M2CD resonances in the presence of AK59 at a drug:tetramer ratio of 2.5:1 inside the incubation solution of 300 µM drug concentration and UBALT09 at a drug:tetramer molar ratio 50:1 inside the incubation solution of 6 mM drug concentration. (A) (H)NH spectrum of WT M2 with AK59 (red) and UBALT09 (blue). (B) ^{15}N-$^{13}C\alpha$ projection of the (H)CαNH spectrum of WT M2 with AK59 (red) and UBALT09 (blue). The samples were reconstituted in DPhPC bilayers at pH 7.8 using a lipid:tetramer molar ratio of 25:1. Both spectra were recorded at pH 7.8 after relaxing the system at acidic pH 3.5 in the presence of the drug and switching back to alkaline pH. All spectra were recorded on an 800 MHz Bruker spectrometer with a 3-channel 1.3 mm probe at 55 kHz MAS. Residue labels of the dimer-of-dimers structure are colored black for chain A and green for chain B.

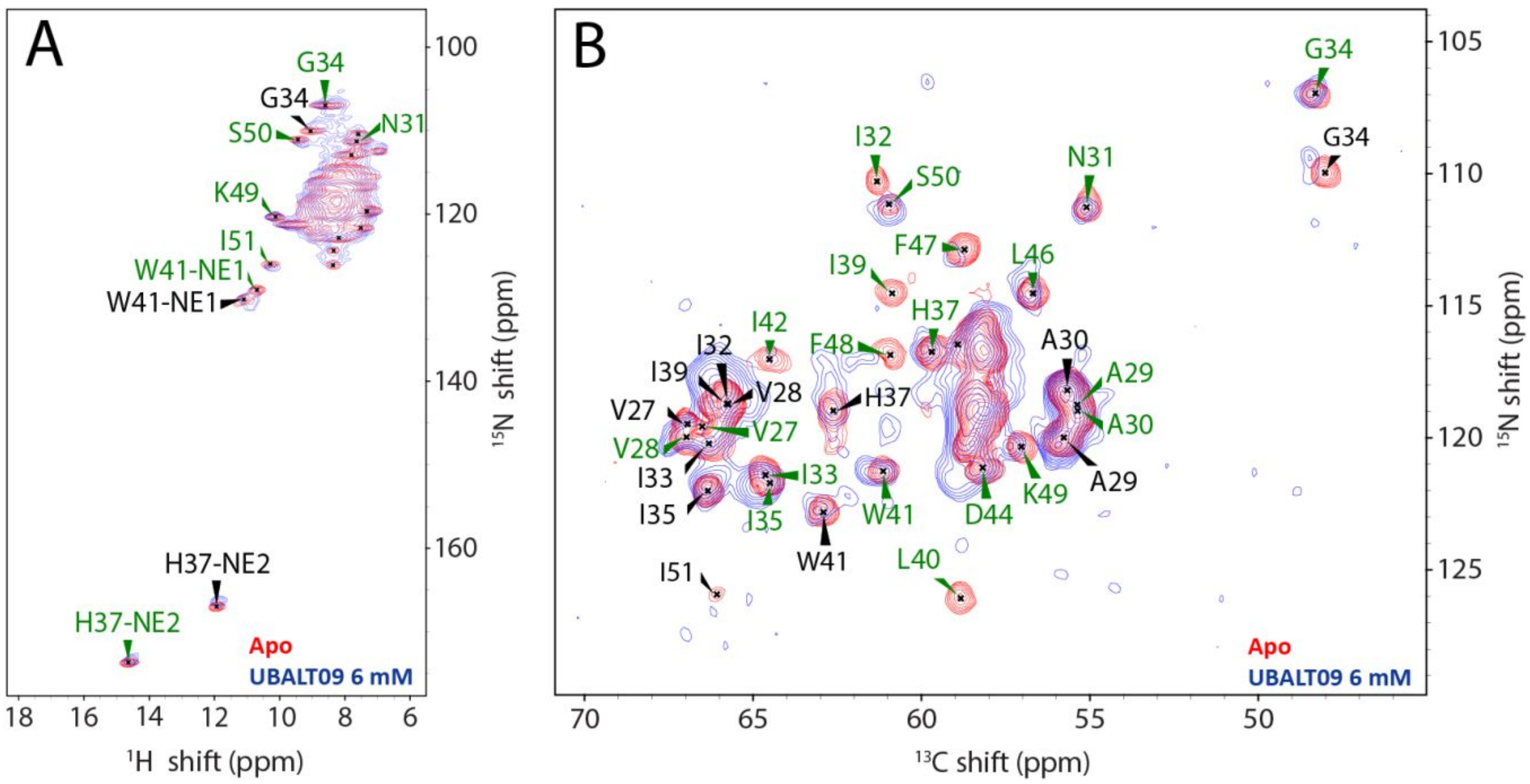

Figure S5. Monitoring the ^{1}H, ^{15}N, and ^{13}C chemical shift perturbation in S31N M2CD resonances in the presence of UBALT09 at a drug:tetramer molar ratio 50:1 inside the incubation solution of 6 mM drug concentration. (A) (H)NH spectrum of S31N M2 with (blue) and without UBALT09 (red). (B) ^{15}N-$^{13}C\alpha$ projection of the (H)CαNH spectrum of S31N M2 with (blue) and without UBALT09 (red). The samples were reconstituted in DPhPC bilayers at pH 7.8 using a lipid:tetramer molar ratio of 25:1. Both spectra were recorded at pH 7.8 after relaxing the system at acidic pH 3.5 in the presence of the drug and switching back to alkaline pH. All spectra were recorded on an 800 MHz Bruker spectrometer with a 3-channel 1.3 mm probe at 55 kHz MAS. Residue labels of the dimer-of-dimers structure are colored black for chain A and green for chain B.

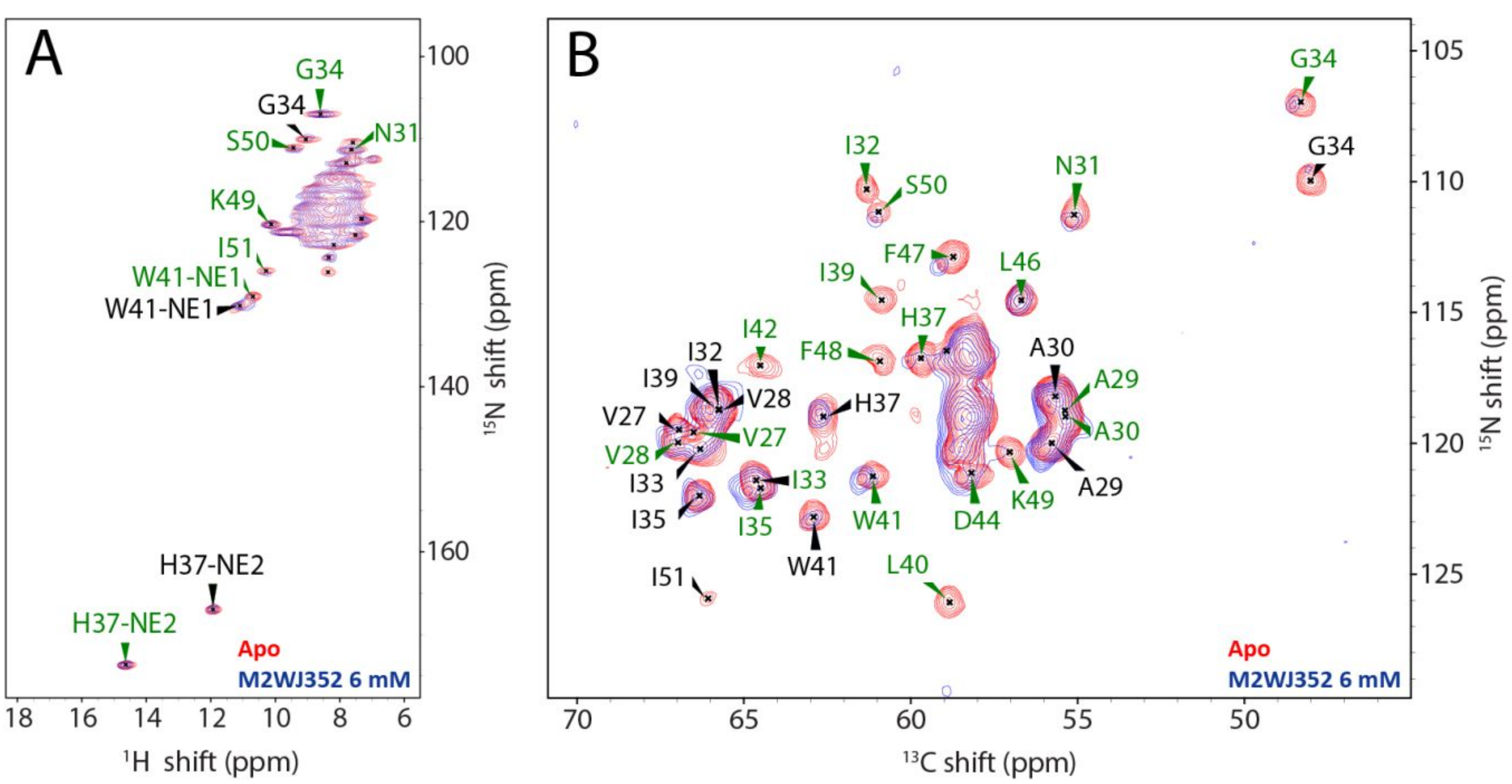

Figure S6. Monitoring the ^{1}H, ^{15}N, and ^{13}C chemical shift perturbation in S31N M2CD resonances in the presence of M2WJ352 at a drug:tetramer molar ratio of 50:1 inside the incubation solution of 6 mM drug concentration. (A) (H)NH spectrum of S31N M2 with (blue) and without M2WJ352 (red). (B) ^{15}N-$^{13}C\alpha$ projection of the (H)CαNH spectrum of S31N M2 with (blue) and without M2WJ352 (red). The samples were reconstituted in DPhPC bilayers at pH 7.8 using a lipid:tetramer molar ratio of 25:1. Both spectra were recorded at pH 7.8 after relaxing the system at acidic pH 3.5 in the presence of the drug and switching back to alkaline pH. All spectra were recorded on an 800 MHz Bruker spectrometer with a 3-channel 1.3 mm probe at 55 kHz MAS. Residue labels of the dimer-of-dimers structure are colored black for chain A and green for chain B.

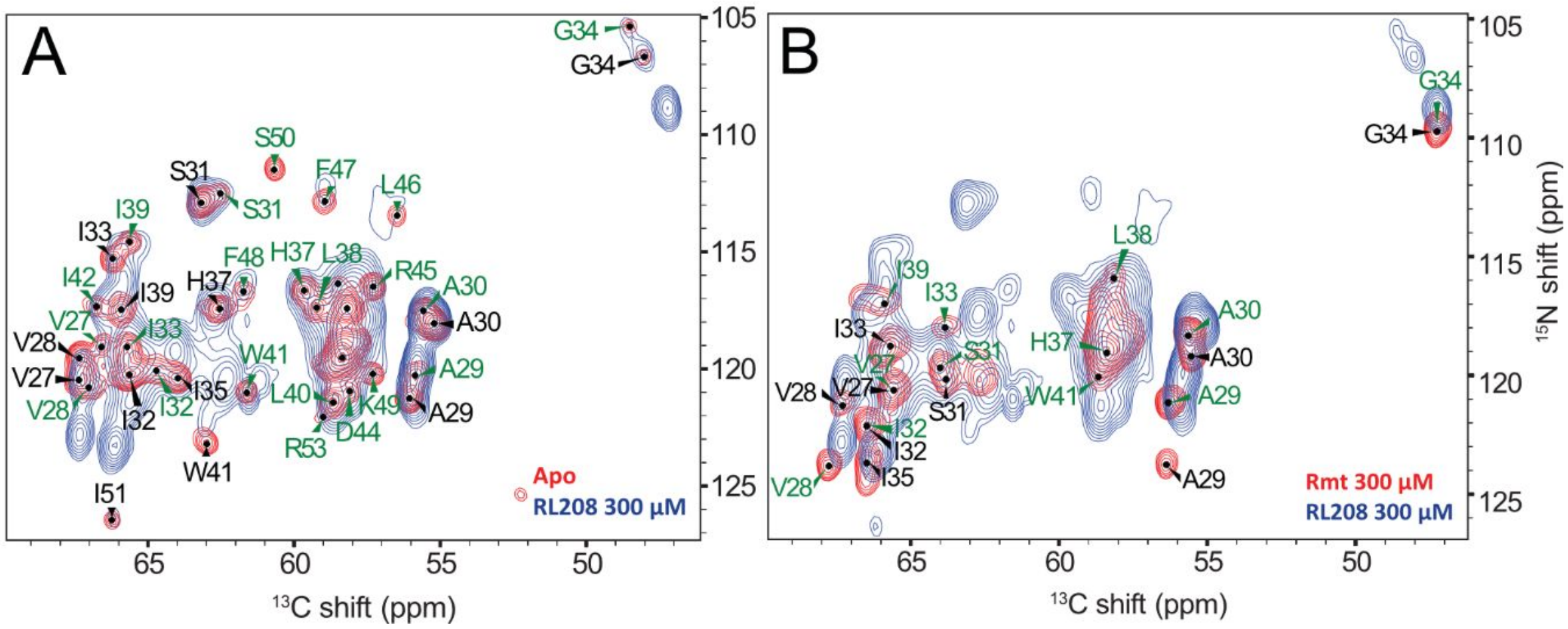

Figure S7. Monitoring the ^{1}H, ^{15}N, and ^{13}C chemical shift perturbation in WT M2CD resonances in the presence of RL208 at a drug:tetramer molar ratio of ratio of 2.5:1 inside the incubation solution of 300 µM drug concentration. (A) ^{15}N-$^{13}C\alpha$ projection of the (H)CαNH spectrum of WT M2 with (blue) and without RL208 (red). (B) ^{15}N-$^{13}C\alpha$ projection of the (H)CαNH spectrum of WT M2 with RL208 (blue) and with Rmt (red). The samples were reconstituted in DPhPC bilayers at pH 7.8 using a lipid:tetramer molar ratio of 25:1. The spectrum of RL208 bound to WT M2CD was captured after storage of the sample for two years, since initially didn't show binding of RL208. This indicates the very slow binding kinetics of the ligand. All spectra were recorded on an 800 MHz Bruker spectrometer with a 3-channel 1.3 mm probe at 55 kHz MAS. Residue labels of the dimer-of-dimers structure are colored black for chain A and green for chain B.

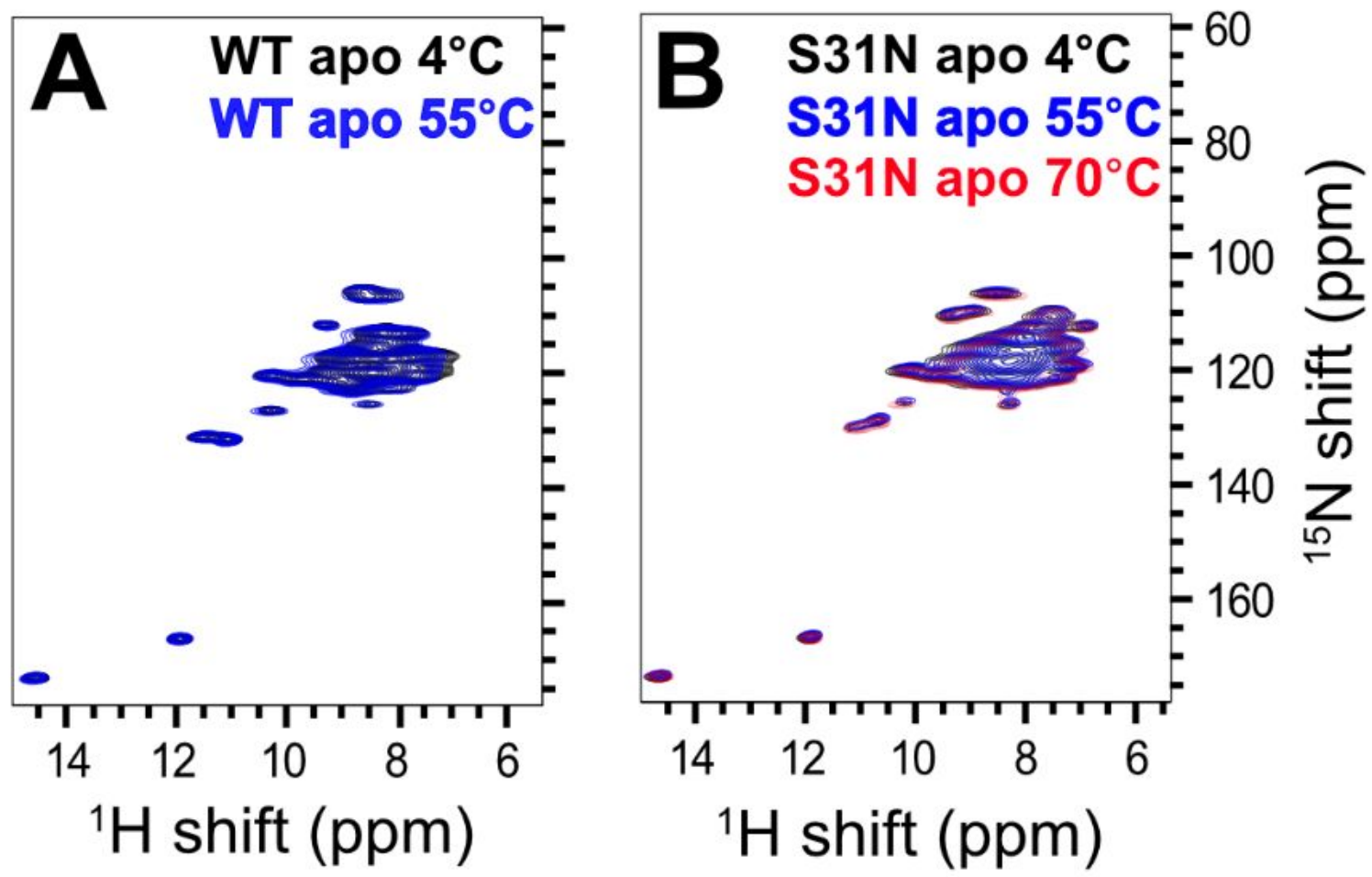

Figure S8. (H)NH spectra of apo WT (A) and S31N M2CD (B) measured at room temperature, after incubation at 4 °C (black), 55 °C (blue) or 70 °C (red) for 2-5 hours. The samples were reconstituted in DPhPC bilayers at pH 7.8 using a lipid:tetramer molar ratio of 25:1. They were incubated in a solution of 300 µM RL208 for WT and UBALT09 for S31N. The drugs did not show binding and the apo form was maintained.

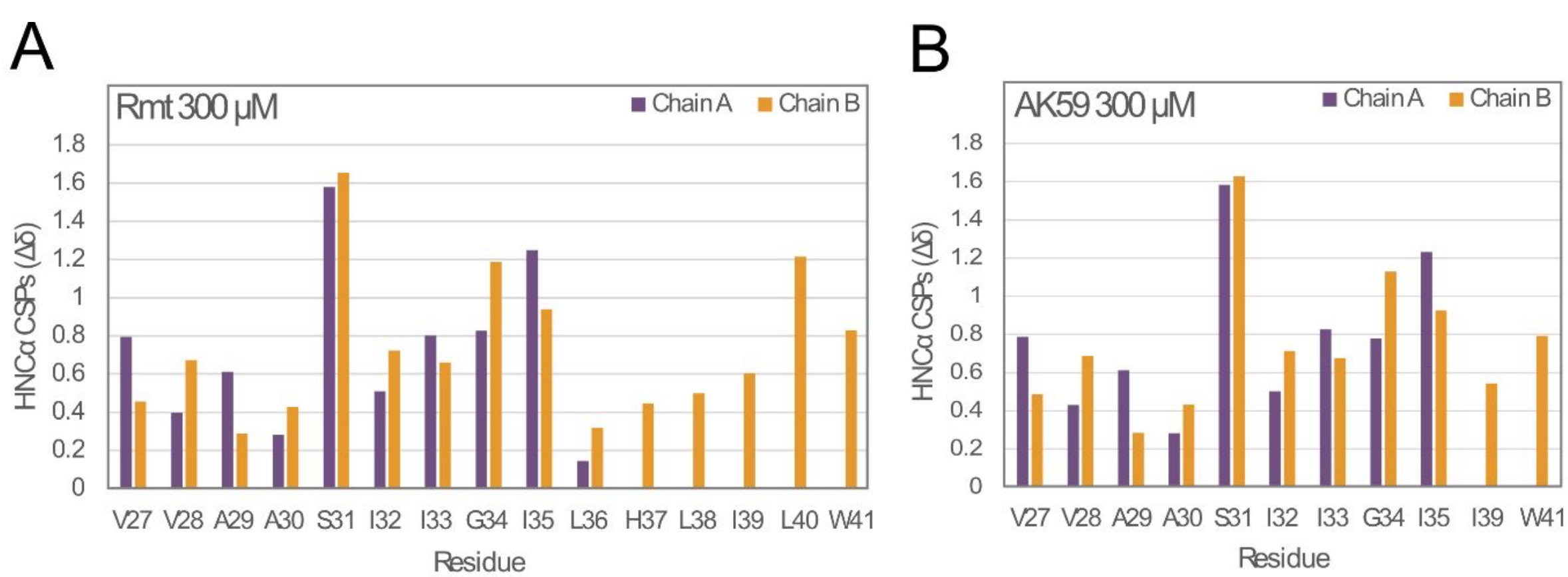

Figure S9. Comparison of chemical shift perturbation (CSP) observed for bound M2 with Rmt (A) and AK59 300 µM (B). Δδ CSPs are shown in purple and yellow for residues in chain A and B, respectively. For AK59 bound M2, residues L36, H37, L38, L40 were either overlapping or not detected due to low sensitivity. The CSPs were calculated for each residue as described by M. P. Williamson. [6]

Simulations

Docking calculations

Compared to the GOLD program with the scoring function Gold Score [7], the Glide program with the scoring function Glide XP [8] produced a higher proportion of docking poses of the compounds having the ammonium group oriented inward inside the WT M2CD [9] and of

M2WJ352 and UBALT09 with the ammonium group oriented outward inside the S31N M2CD [10] in agreement with the experimental results [10, 11] (Figure 6, 7). The combination of Glide with the induced fit [12] method produced the best fit. The M2CD complexes were embedded in lipid buffers and subjected to MD simulations.

Measurements in the MD simulations

We carried out MD simulations in the complexes of WT or S31N M2CD correspondingly with Rmt enantiomers (R-Rim, S-Rim), AK59, RL208, M2WJ352, and UBALT09 at pH 7.8, with M2CD adopting the closed conformation at the C-terminal side of the membrane [11] (His37-tetrad charge 0). The M2CD (H37 Nε) tetramer was embedded in 13 Å lipid buffer. These bilayers include ca. 130 – 140 lipids surrounding M2CD. We applied for the simulations the CHARMM36m force field. [9, 13]

We investigated:

A) The blockage/passage of bulk waters from the Val27 gate in the WT and S31N M2CD pore in the apo-protein and in complex with an inhibitor and the orientation of the ligand inside the M2CD pore.
B) The stability of the M2–ligand complexes and the location of the blockers inside the M2CD pore. The location of the pore blockers depends on the conformation of M2CD and their interactions with the M2TM.
C) The H–bonds between the four His37 in the apo-protein WT M2CD and between ligand and waters, Ala30 or Asn31 (in S31N M2CD complexes).

Methods

Computational biochemistry methods

Protein Structure Preparation

Structures of the Rim, AK59, RL208, M2WJ352, and UBALT09 were sketched with the Maestro program and model-built with the Schrödinger 2021-2 platform (Schrödinger Release 2021-2: Maestro, Schrödinger, LLC, New York, NY, 2021; Maestro-Desmond Interoperability Tools, Schrödinger, New York, NY, 2021). The models were minimized using the conjugate gradient method, MMFF94 force field [15], and distance-dependent dielectric constant 4.0 until a convergence threshold of 2.4×10^{-5} kcal mol^{-1} $Å^{-1}$ was reached. The R–Rim, S–Rim, AK59, RL208, M2WJ352, and UBALT09 have a charged ammonium group at pH 7. The WT M2TM was modeled using the PDB ID 2KQT [16] structure in which Amt is complexed. The WT M2CD was modeled using the PDB ID 2L0J structure of WT M2CD apoprotein. [17] Previous PMF calculations [18] using the W41 χ2 dihedral as the reaction coordinate showed that the energetically favored conformation for Trp41 in AH M2 is t-90 instead of t-105 observed in the PDB ID 2L0J structure. [17] Accordingly, we set the W41 χ2 dihedrals to 90°. To form the M2CD–Amt complex, the heavy atoms of the TM helices

between M2CD (PDB ID 2L0J) [17] and M2TM–Amt complex (PDB ID 2KQT) [16] were superimposed (root-mean-square deviation (RMSD) < 0.1 Å), and the M2TM was deleted. The protein preparation module in Schrödinger's 2021-2 Maestro (Schrödinger Release 2021-2: Maestro, Schrödinger, LLC, New York, NY, 2021-2; Maestro- Desmond Interoperability Tools, Schrödinger, New York, NY, 2021) was used to add missing hydrogen atoms. N- and C-termini of the M2 model systems were capped by acetyl and methylamino groups, respectively. The histidine residues (His37 and His57) were protonated at the Nε site according to experimental data. [19-22] All hydrogens of the protein complex were minimized using the OPLS2005 force field [23-25] and distance-dependent dielectric constant 4.0, with the conjugate gradient method until the RMSD of potential energy reached 2.4×10^{-5} kcal mol^{-1} $Å^{-1}$ by means of Maestro/Macromodel. [26] Geometries of the complexes were energy-minimized in the gas phase with OPLS2005 [23-25] using the conjugate gradient method and a distance-dependent dielectric constant of 4 Å until a convergence value of 0.01 kcal mol^{-1} $Å^{-1}$ was reached.

MD simulations trajectories analysis

The analysis of the trajectories was performed with Desmond software, (Schrödinger Release 2021-1: Desmond Molecular Dynamics System, D. E. Shaw Research, New York, NY, 2021. Maestro-Desmond Interoperability Tools, Schrödinger, New York, NY, 2021). The Desmond trajectories (.cms) were viewed and analyzed within the Maestro interface. The RMSDs of Cα carbons and heavy atoms of ligand were calculated with modules using Schrödinger's python3 interpreter. A representative structure of the whole MD simulation was generated for each system using the average structure panel in Maestro and the RMSD plots were generated using python3.10 (matplotlib module). For the calculation of specific distances within MD simulation, a python3 script (monitor_asl.py) was used to monitor each residue. Distance measurements were performed between the COM of the residues Val27, Ala30, and Gly34 and the COM of the ligand's cage and between the COM of the His37 and the ligands charged amine group (N+). Also using the Simulation Event Analysis panel from Maestro, the number of Hydrogen bonds between each ligand and the water molecules and Ala30 was monitored.

Stability of the M2CD–ligand complexes and location of the molecules

The RMSD(Ca) and RMSD(ligand) are shown in Figure S10; as is shown the systems reached equilibration inside the 250 ns-MD simulations.

We measured distances between the ligand and some of the TM residues. Table S3 shows the distance between the center of mass (COM) of the Val27, Ala30, and Gly34 tetrad and the COM of the cage alkyl of each ligand in the 250 ns MD simulations. The MD simulations of Rim enantiomers in complex with M2CD suggested that both enantiomers adopt the same position inside the M2 channel pore. This agrees with the thermodynamic and kinetic data showing that they have identical binding free energy, channel blocking efficiency, and

k_{on} /k_{off} rate constants measured by EP, as well as similar in vitro antiviral potencies against influenza A virus. [27, 28]

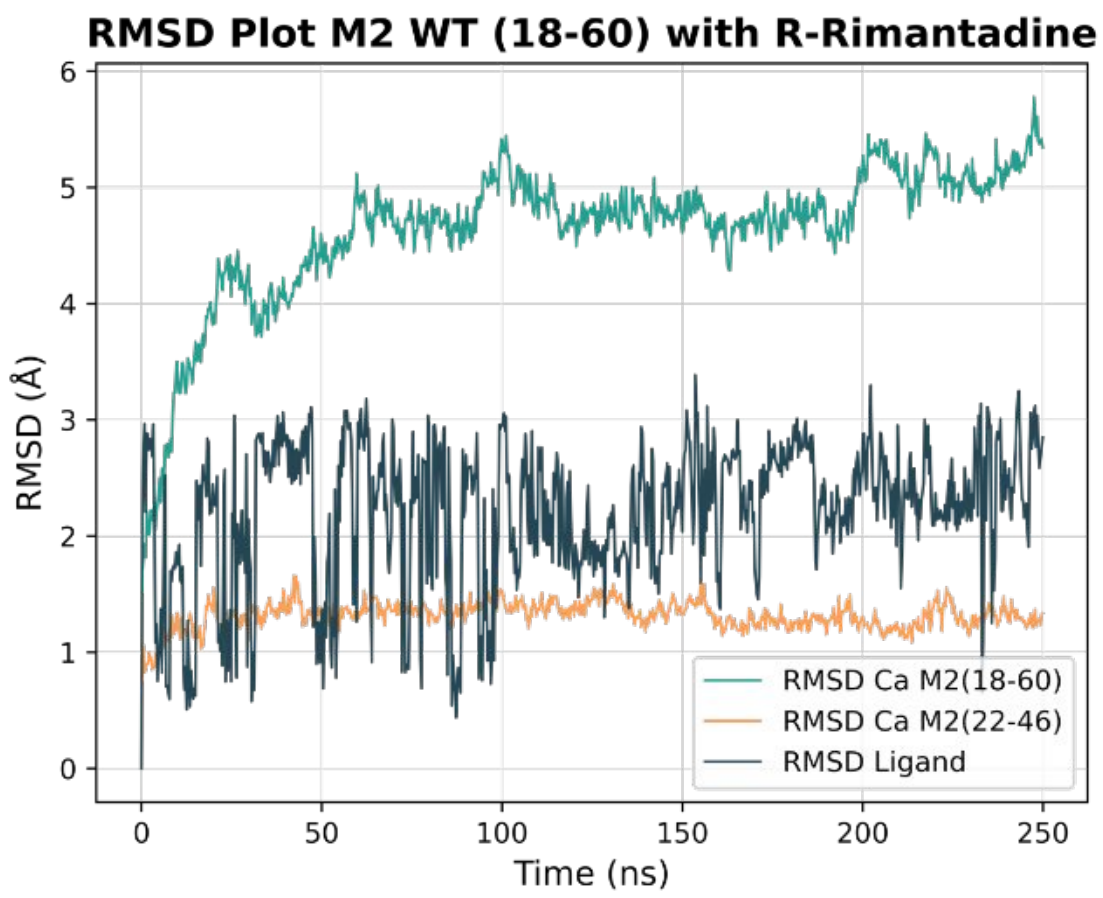

Figure S10. RMSD(Ca) and RMSD(ligand) in the MD simulations of the apo-protein and in complex with ligands during the 250ns-MD simulations with CHARMM36m force field.

Table S3. Distance (in Å) between the COM of the ligand's cage and the COM of the four Val27, Ala30, Gly34 or between COM of His37 and ligand's N+ from 250 ns MD simulation with CHARMM36m forcefield.

Molecule	Type	COM cage – COM Val27	COM cage – COM Ala30	COM cage – COM Gly34	COM His37 - N+ ligand
R–Rim	WT	4.87 ± 0.27	0.56 ± 0.22	5.11 ± 0.30	7.20 ± 0.32
S–Rim	WT	4.85 ± 0.28	0.58 ± 0.23	5.12 ± 0.31	7.18 ± 0.32
AK59	WT	5.16 ± 0.24	0.38 ± 0.16	4.80 ± 0.26	7.08 ± 0.26
RL208	WT	4.67 ± 0.41	0.87 ± 0.37	5.52 ± 0.46	6.98 ± 0.49
M2WJ352	WT	4.29 ± 0.36	1.07 ± 0.36	5.53 ± 0.41	-
UBALT09	WT	4.37 ± 0.41	0.971 ± 0.39	5.32 ± 0.44	-
R–Rim	S31N	5.90 ± 0.39	0.53 ± 0.29	4.40 ± 0.39	-
S–Rim	S31N	5.86 ± 0.50	0.59 ± 0.34	4.33 ± 0.51	-
AK59	S31N	6.01 ± 0.42	0.68 ± 0.30	3.90 ± 0.45	-
RL208	S31N	6.74 ± 0.53	1.32 ± 0.52	3.43 ± 0.56	-
M2WJ352	S31N	6.76 ± 0.26	1.20 ± 0.25	3.59 ± 0.27	-
UBALT09	S31N	6.47 ± 0.29	0.98 ± 0.28	3.84 ± 0.31	-

Hydrogen bonds between the ligand and water, and between the ligand and the residue 30 or 31 tetrad, were counted with GROMACS gmx hbond using default geometric criteria (0.35 nm donor–acceptor distance, 30° cutoff). The results are shown in Table S4.

MD analysis of AK59 and UBALT09 in POPC and DPhPC lipids, including protonated or non-protonated His37

Systems

No	Ligand	Lipid	Protonation	M2 Type
1	AK59	POPC, DPhPC	H37-ε and H37-p	WT and S31N
2	UBALT09	POPC, DPhPC	H37-ε and H37-p	WT and S31N

H37-ε: neutral
H37-p: protonated

Table S4. H–bonds analysis from the 250 ns simulations of protein–ligand complexes of WT/S31N M2CD in CHARMM36m force field.

Molecule	Bilayer	Protonation	Type	Ligand - waters H-bonds	Ligand - A30 H-bonds	Ligand - N31 H-bonds
R–Rim	POPC	H37-ε	WT	2.98 ± 0.33	0.00 ± 0.05	-
S–Rim	POPC	H37-ε	WT	3.00 ± 0.24	0.00 ± 0.00	-
AK59	POPC	H37-ε	WT	2.99 ± 0.13	0.00 ± 0.00	-
RL208	POPC	H37-ε	WT	2.97 ± 0.42	0.00 ± 0.00	-
M2WJ352	POPC	H37-ε	WT	2.09 ± 0.35	0.02 ± 0.03	-
UBALT09	POPC	H37-ε	WT	2.12 ± 0.44	0.00 ± 0.00	-
R–Rim	POPC	H37-ε	S31N	1.02 ± 0.43	-	1.93 ± 0.28
S–Rim	POPC	H37-ε	S31N	1.08 ± 0.52	-	1.90 ± 0.34
AK59	POPC	H37-ε	S31N	1.07 ± 0.43	-	1.93 ± 0.29
RL208	POPC	H37-ε	S31N	1.38 ± 0.65	-	1.54 ± 0.52
M2WJ352	POPC	H37-ε	S31N	0.41 ± 0.57	-	2.59 ± 0.70
UBALT09	POPC	H37-ε	S31N	0.84 ± 0.68	-	2.21 ± 0.75
AK59	DPhPC	H37-ε	WT	1.91 ± 0.29	0.01 ± 0.11	–

AK59	DPhPC	H37-p	WT	0.92 ± 1.01	0.06 ± 0.24	–
AK59	POPC	H37-p	WT	1.05 ± 0.98	0.11 ± 0.31	–
AK59	DPhPC	H37-ε	S31N	1.03 ± 0.25	–	1.86 ± 0.40
AK59	DPhPC	H37-p	S31N	1.03 ± 0.26	–	1.91 ± 0.32
AK59	POPC	H37-p	S31N	1.17 ± 0.48	–	1.73 ± 0.56
UBALT09	DPhPC	H37-ε	WT	1.17 ± 0.83	0.00 ± 0.05	–
UBALT09	DPhPC	H37-p	WT	1.54 ± 0.50	0.00 ± 0.00	–
UBALT09	POPC	H37-p	WT	1.27 ± 0.95	0.00 ± 0.00	–
UBALT09	DPhPC	H37-ε	S31N	0.02 ± 0.13	–	3.04 ± 0.75
UBALT09	DPhPC	H37-p	S31N	0.43 ± 0.69	–	2.69 ± 1.17
UBALT09	POPC	H37-p	S31N	0.39 ± 0.62	–	2.54 ± 0.93

Pore profiles were computed with HOLE2 through the mdahole2 package for MDAnalysis, with the channel vector fixed to the membrane normal and the starting point at the His37 tetrad. The minimum radius was taken between residues 22 and 46 of M2.

Table S5. Pore radius analysis from the 250 ns simulations of protein–ligand complexes of WT/S31N M2CD in different lipids and His37 protonation states.

Ligand	Type	Protonation	Lipid	Min radius (Å)
AK59	WT	H37-ε	DPhPC	0.55 ± 0.22
AK59	WT	H37-ε	POPC	0.50 ± 0.18
AK59	WT	H37-p	DPhPC	**0.94 ± 0.16**
AK59	WT	H37-p	POPC	**0.94 ± 0.17**
AK59	S31N	H37-ε	DPhPC	0.68 ± 0.52
AK59	S31N	H37-ε	POPC	1.03 ± 0.45
AK59	S31N	H37-p	DPhPC	**1.75 ± 0.46**
AK59	S31N	H37-p	POPC	**1.53 ± 0.26**
UBALT09	WT	H37-ε	DPhPC	0.93 ± 0.23

UBALT09	WT	H37-ε	POPC	0.75 ± 0.30
UBALT09	WT	H37-p	DPhPC	**0.82 ± 0.17**
UBALT09	WT	H37-p	POPC	**0.89 ± 0.25**
UBALT09	S31N	H37-ε	DPhPC	0.68 ± 0.52
UBALT09	S31N	H37-ε	POPC	0.38 ± 0.29
UBALT09	S31N	H37-p	DPhPC	**1.38 ± 0.34**
UBALT09	S31N	H37-p	POPC	**1.46 ± 0.35**

Mean of the per-frame minimum radius ± standard deviation, taken between residues 22 and 46. The ligand is excluded from the calculation, so these describe the protein's own conformation.

Table S6. Mean of the per-frame minimum radius from the 250 ns simulations of protein–ligand complexes of WT/S31N M2CD.

**Effect of His37 protonation**						**Effect of the S31N mutation**					
Ligand	Type	Lipid	H37-ε	H37-p	Change	Ligand	Protonation	Lipid	WT	S31N	Direction
AK59	WT	DPhPC	0.55	0.94	+0.39	AK59	H37-p	DPhPC	0.94	1.75	wider
AK59	WT	POPC	0.50	0.94	+0.44	AK59	H37-p	POPC	0.94	1.53	wider
AK59	S31N	DPhPC	0.68	1.75	+1.07	AK59	H37-ε	DPhPC	0.55	0.68	wider
AK59	S31N	POPC	1.03	1.53	+0.50	AK59	H37-ε	POPC	0.50	1.03	wider
UBALT09	WT	DPhPC	0.93	0.82	**−0.11**	UBALT09	H37-p	DPhPC	0.82	1.38	wider
UBALT09	WT	POPC	0.75	0.89	+0.14	UBALT09	H37-p	POPC	0.89	1.46	wider
UBALT09	S31N	DPhPC	0.68	1.38	+0.70	UBALT09	H37-ε	DPhPC	0.93	0.68	**narrower**
UBALT09	S31N	POPC	0.38	1.46	+1.08	UBALT09	H37-ε	POPC	0.75	0.38	**narrower**

Protonating His37 widens the pore. Seven of eight matched comparisons widen the pore, by 0.14 to 1.08 Å. The one exception is UBALT09/WT/DPhPC. The four His37 side chains carry positive charge in the H37-p state, and mutual electrostatic repulsion within the tetrad forces them apart, splaying the helix bundle. The channel that results is more open at its narrowest point.

Water permeation and pore occlusion in WT and S31N influenza A M2

Water traversal of the His37 gate

Every water oxygen was tracked by atom index for the whole trajectory. The channel axis was taken as the vector from the residue 22 center of mass to the residue 41 center of mass, recomputed each frame, and each water was projected onto it, giving a signed coordinate measured from the His37 tetrad center of mass.

A water was counted as having traversed the gate only if all of the following conditions were met:

- it moved from the residue 22 side to the residue 41 side, the physiological direction; reverse motion was tallied separately and never counted;
- it lay within 0.6 nm of the channel axis at both ends of the step;
- it lay within 1.5 nm of the gate plane at both ends of the step, so that a molecule crossing a periodic boundary far from the gate cannot register;
- it moved less than 2 nm in that step, discarding unphysical jumps;
- it reached at least 0.5 nm beyond the gate on the residue 22 side and later at least 0.5 nm beyond it on the residue 41 side, within one continuous visit, so that a molecule resting at the plane and jittering across it does not qualify;
- it was not within 5 Å of the His37 tetrad or of the ligand in the first frame; such waters were placed during equilibration, their provenance is unknown, and they were barred permanently;
- it had not already been counted; each molecule contributes at most one traversal.

For all periodic-boundary handling, MDAnalysis minimize vectors were used with the box being re-read at every frame, since the cell fluctuates under constant pressure.

Table S7. Analysis of the number of waters traversing the His37 gate during the 250 ns simulations.

AK59

Lipid	Protonation	WT (n[a])	WT %[b]	S31N (n[a])	S31N %[b]
POPC	H37-ε	**2**	0.024	**32**	0.307
DPhPC	H37-ε	**5**	0.055	**28**	0.315
POPC	H37-p	47	0.581	148	1.850
DPhPC	H37-p	32	0.355	180	2.017

Lipid	Protonation	WT (n)	WT %	S31N (n)	S31N %
POPC	H37-ε	**170**	1.330	**32**	0.309
DPhPC	H37-ε	**80**	0.902	**63**	0.708
POPC	H37-p	110	1.395	197	2.460
DPhPC	H37-p	32	0.359	125	1.402

[a] Distinct water molecules traversing the His37 gate from residue 22 to residue 41, each counted once.
[b] “Waters %” expresses that count as a percentage of all water in that system, computed from the trajectory alone.

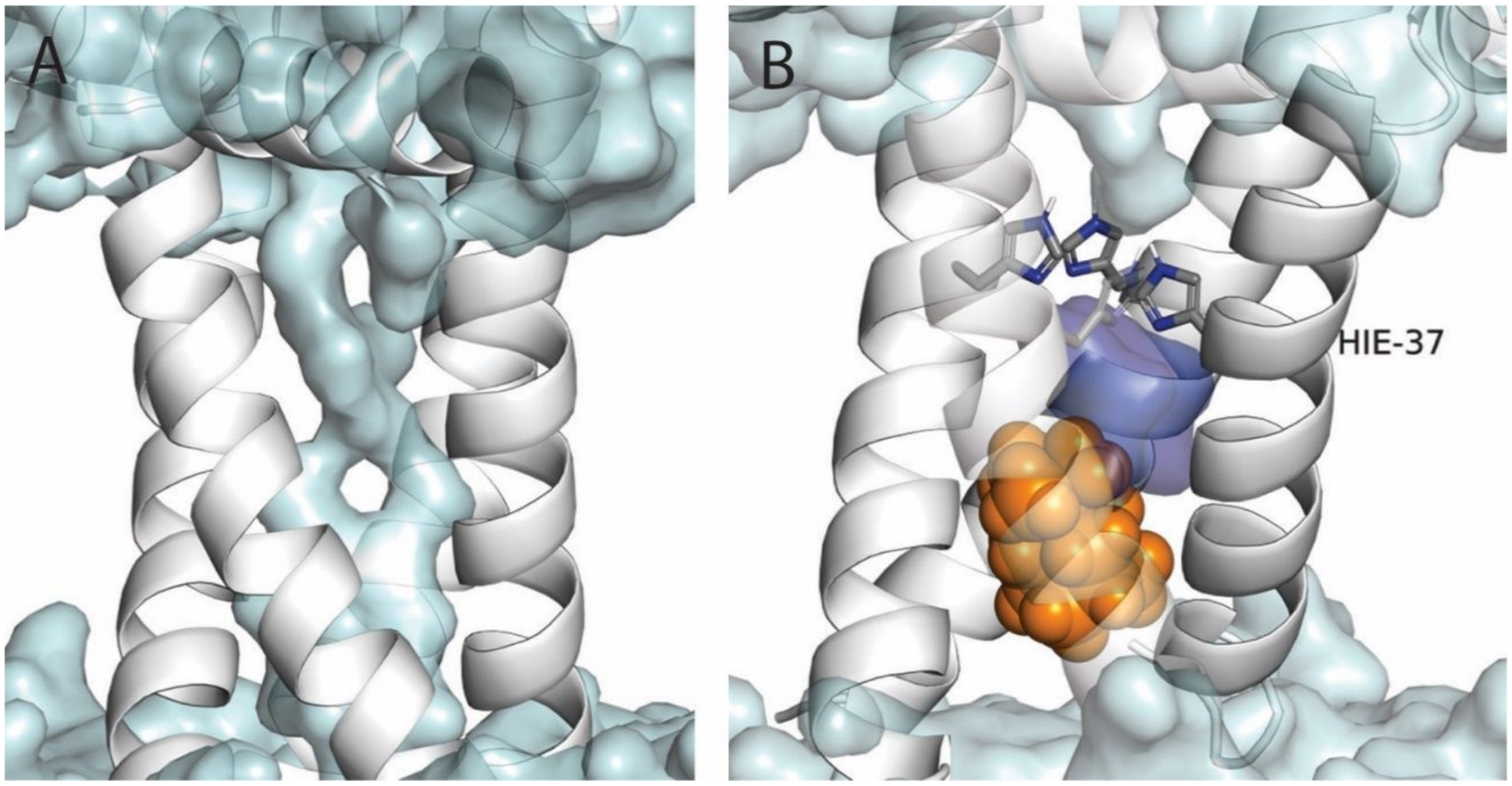

Figure S11. Water density inside the pore is shown in WT M2CD (PDB ID 2L0J) in 13 Å lipid buffer. (A) WT M2CD apoprotein, there is free flow of water molecules within the TM pore. (B) WT M2CD in complex with a ligand shows the blockage of the water flow within the TM pore and the trapped water molecules between ligand and His37 (HIE). Ligand atoms are shown in Van der Waals spheres colored orange, the trapped water molecules are shown in blue color in surface representation, and with cyan color the rest of water molecules in surface representation as well. The WT M2CD is shown with cartoon representation in white color and the His37 of the TM domain with stick representation in deep gray color.

Liposomal proton flux assay

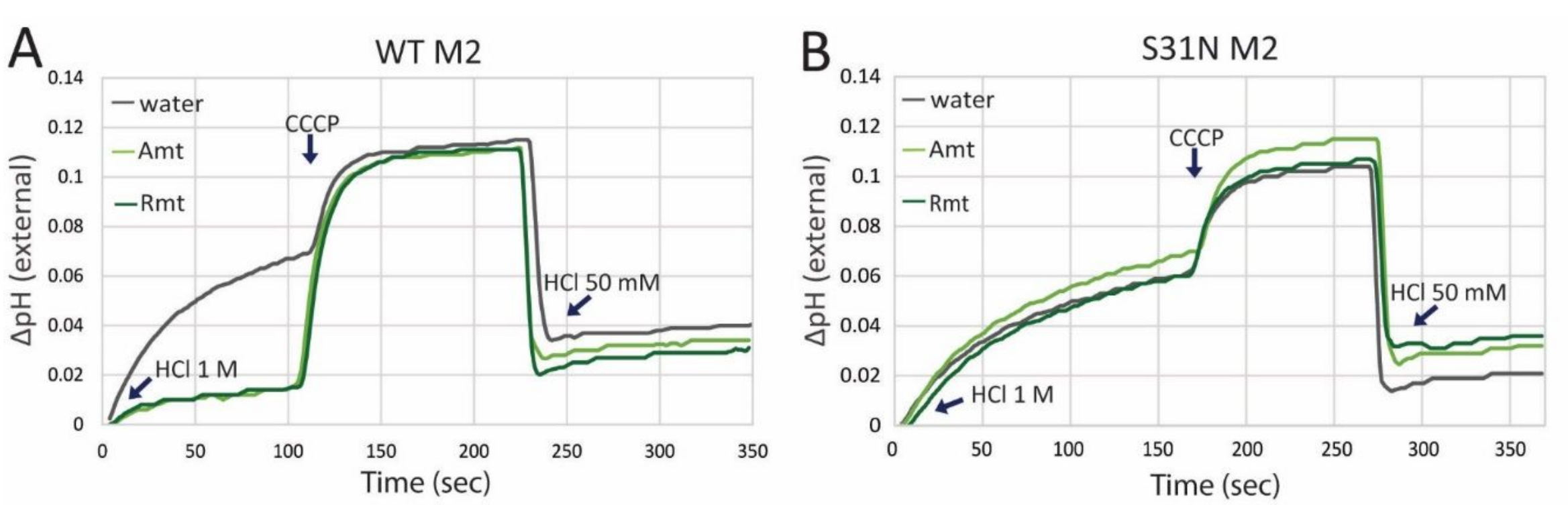

Figure S12. Liposomal proton flux assay applied to WT and S31N M2CD in the presence of Amt, Rmt. The 5-point moving average method was used to smoothen the data points of

the curves. Time zero corresponds to the pH equilibration of the external solution at pH ~ 6.3 after the addition of 1 M HCl. M2CD activation and proton translocation into the liposome interior is shown as an increase of ΔpH. Addition of proton uncoupler CCCP terminates the experiment. The buffering capacity of the system is evaluated by adding 50 mM HCl. (A) Proton conduction of WT M2CD is strongly inhibited by 50 μM of Amt, Rmt. (B) Proton conduction of S31N M2CD is not inhibited by any of the drugs tested. The measurement window was extended by 1 min after the first HCl addition.

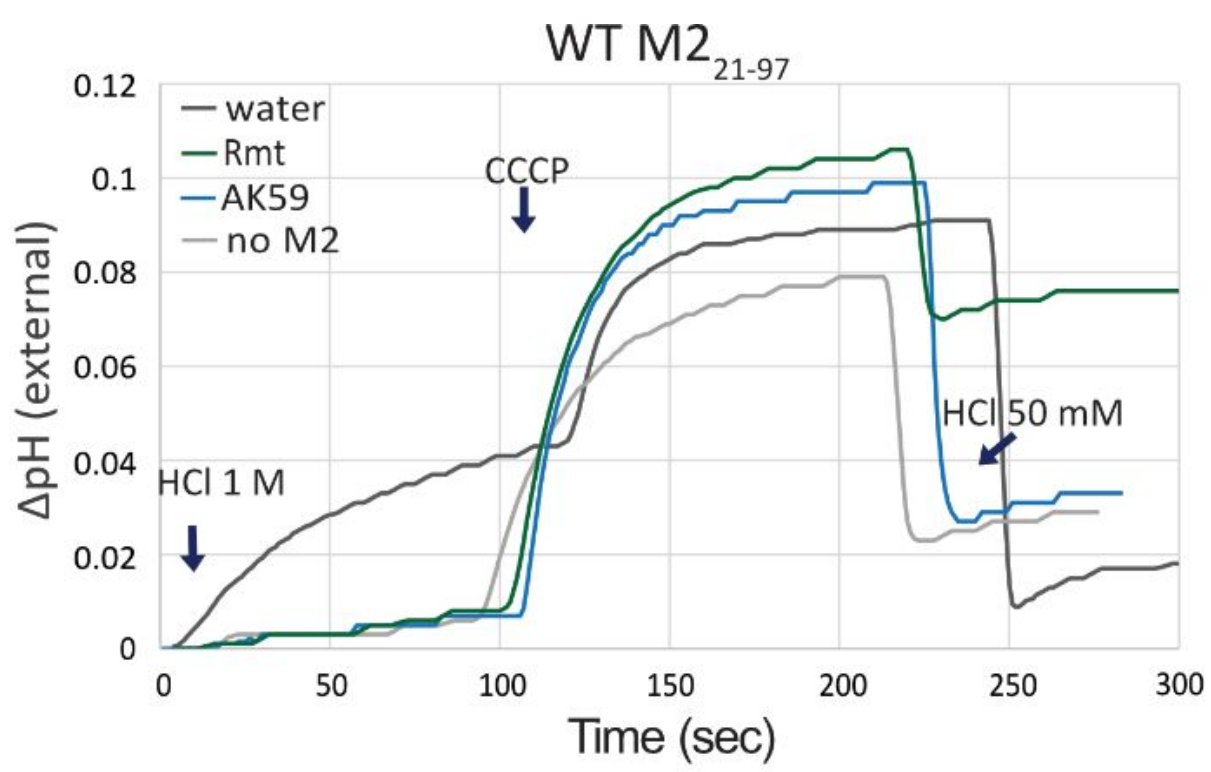

Figure S13. Liposomal proton flux assay applied to WT_{21-97} in the presence of AK59, Rmt and to liposomes that carry no M2. The comparison of the last with the WT inhibitors shows that the liposomes are not perfectly sealed, which explains the slight increase of the external pH in these three cases.

Table S8. Proton conduction rates in protons/sec/tetramer[a]

	WT_{18-60}	WT_{21-97}
H_2O	1.6 ± 0.2	1.9 ± 0.2
Rmt	0.2 ± 0.03	0.4 ± 0.1
AK59	0.3 ± 0.4	0.3 ± 0.2
RL208	1.1 ± 0.1	0.6 ± 0.2
UBALT09	1.6 ± 0.3	2.2 ± 0.2
M2WJ352	1.6 ± 0.4	2.0 ± 0.4

[a]average values from two independent experiments

Protein sequences

*note that C19 and C50 were changed to S to avoid issues with disulfide bond formation. In the 21-97 protein, H57 and H90 were changed to Q, as in previous work. [29] H57 is not essential to the virus, and H90 lies far removed from the channel function of the protein, squarely in the cytoplasmic residues that interact with M1. [30]

WT_{18-60} A Udorn/307/1972 (H3N2)

^{18}RSNDSSDPLV^{27} ^{28}VAASIIGILH^{37} ^{38}LILWILDRLF^{47} ^{48}FKSIYRFFEH^{57} ^{58}GLK^{60}

S31N$_{18-60}$ A Udorn/307/1972 (H3N2)

^{18}RSNDSSDPLV^{27} ^{28}VAA**N**IIGILH^{37} ^{38}LILWILDRLF^{47} ^{48}FKSIYRFFEH^{57} ^{58}GLK^{60}

WT$_{21-97}$ A Udorn/307/1972 (H3N2)

^{21}DSSDPLV^{27} ^{28}VAASIIGILH^{37} ^{38}LILWILDRLF^{47} ^{48}FKSIYRFFEQ^{57}
^{58}GLKRGPSTEG^{67} ^{68}VPESMREEYR^{77} ^{78}KEQQSAVDAD^{87} ^{88}DSQFVSIELE^{97}

References

(1) Drakopoulos, A.; Tzitzoglaki, C.; Ma, C.; Freudenberger, K.; Hoffmann, A.; Hu, Y.; Gauglitz, G.; Schmidtke, M.; Wang, J.; Kolocouris, A. Affinity of Rimantadine Enantiomers against Influenza A/M2 Protein Revisited. *ACS Medicinal Chemistry Letters* **2017**, *8*, 145–150. DOI: 10.1021/acsmedchemlett.6b00311.

(2) Stampolaki, M.; Hoffmann, A.; Tekwani, K.; Georgiou, K.; Tzitzoglaki, C.; Ma, C.; Becker, S.; Schmerer, P.; Döring, K.; Stylianakis, I.; et al. A Study of the Activity of Adamantyl Amines against Mutant Influenza A M2 Channels Identified a Polycyclic Cage Amine Triple Blocker, Explored by Molecular Dynamics Simulations and Solid-State NMR. *ChemMedChem* **2023**, e202300182. DOI: 10.1002/CMDC.202300182.

(3) Wang, J.; Wu, Y.; Ma, C.; Fiorin, G.; Wang, J.; Pinto, L. H.; Lamb, R. A.; Klein, M. L.; DeGrado, W. F. Structure and inhibition of the drug-resistant S31N mutant of the M2 ion channel of influenza A virus. *Proceedings of the National Academy of Sciences of the United States of America* **2013**, *110*, 1315–1320. DOI: 10.1073/pnas.1216526110.

(4) Turcu, A. L.; Leiva, R.; Ma, C.; Georgiou, K.; Brea, J. M.; Loza, M. I.; Val, C.; Naesens, L.; Wang, J.; Kolocouris, A.; et al. Exploring polycyclic scaffolds as adamantane replacements in M2 channel inhibitors of Influenza A virus. *bioRxiv* **2025**. DOI: 10.1101/2025.07.26.666854.

(5) Movellan, K. T.; Wegstroth, M.; Overkamp, K.; Leonov, A.; Becker, S.; Andreas, L. B. Real-time tracking of drug binding to influenza A M2 reveals a high energy barrier. *Journal of Structural Biology: X* **2023**, *8*. DOI: 10.1016/j.yjsbx.2023.100090.

(6) Williamson, M. P. Using chemical shift perturbation to characterise ligand binding. *Progress in Nuclear Magnetic Resonance Spectroscopy* **2013**, *73*, 1–16. DOI: 10.1016/j.pnmrs.2013.02.001.

(7) Jones, G.; Willett, P.; Glen, R. C.; Leach, a. R.; Taylor, R. Development and validation of a genetic algorithm for flexible docking. *Journal of molecular biology* **1997**, *267*, 727–748. DOI: 10.1006/jmbi.1996.0897.

(8) Friesner, R. A.; Murphy, R. B.; Repasky, M. P.; Frye, L. L.; Greenwood, J. R.; Halgren, T. A.; Sanschagrin, P. C.; Mainz, D. T. Extra precision glide: Docking and scoring incorporating a model of hydrophobic enclosure for protein-ligand complexes. *Journal of Medicinal Chemistry* **2006**, *49*, 6177–6196. DOI: 10.1021/JM051256O.

(9) Best, R. B.; Zhu, X.; Shim, J.; Lopes, P. E. M.; Mittal, J.; Feig, M.; MacKerell, A. D. Optimization of the additive CHARMM all-atom protein force field targeting improved sampling of the backbone φ, ψ and side-chain χ1 and χ2 Dihedral Angles. *Journal of Chemical Theory and Computation* **2012**, *8*, 3257–3273. DOI: 10.1021/CT300400X.

(10) Ma, C.; Polishchuk, a. L.; Ohigashi, Y.; Stouffer, a. L.; Schon, A.; Magavern, E.; Jing, X.; Lear, J. D.; Freire, E.; Lamb, R. a.; et al. Identification of the functional core of the influenza A virus A/M2 proton-selective ion channel. *Proc Natl Acad Sci U S A* **2009**, *106*, 12283–12288. DOI: 10.1073/pnas.090572610.

(11) Thomaston, J. L.; Polizzi, N. F.; Konstantinidi, A.; Wang, J.; Kolocouris, A.; Degrado, W. F. Inhibitors of the M2 proton channel engage and disrupt transmembrane networks of hydrogen-bonded waters. *Journal of the American Chemical Society* **2018**, 15219–15226. DOI: 10.1021/jacs.8b06741.
(12) Sherman, W.; Day, T.; Jacobson, M. P.; Friesner, R. A.; Farid, R. Novel procedure for modeling ligand/receptor induced fit effects. *Journal of Medicinal Chemistry* **2006**, *49*, 534–553. DOI: 10.1021/JM050540C.
(13) Huang, J.; Mackerell, A. D. CHARMM36 all-atom additive protein force field: Validation based on comparison to NMR data. *Journal of Computational Chemistry* **2013**, *34*, 2135–2145. DOI: 10.1002/JCC.23354.
(14) Tzitzoglaki, C.; Wright, A.; Freudenberger, K.; Hoffmann, A.; Tietjen, I.; Stylianakis, I.; Kolarov, F.; Fedida, D.; Schmidtke, M.; Gauglitz, G.; et al. Binding and Proton Blockage by Amantadine Variants of the Influenza M2WT and M2S31N Explained. *Journal of Medicinal Chemistry* **2017**, *60*, 1716–1733. DOI: 10.1021/ACS.JMEDCHEM.6B01115.
(15) Halgren, T. T. A. Merck molecular force field. I. Basis, form, scope, parameterization, and performance of MMFF94. *Journal of Computational Chemistry* **1996**, *17*, 490–519. DOI: 10.1002/(SICI)1096-987X(199604)17:5/6<490::AID-JCC1>3.0.CO;2-P.
(16) Cady, S. D.; Schmidt-Rohr, K.; Wang, J.; Soto, C. S.; DeGrado, W. F.; Hong, M. Structure of the amantadine binding site of influenza M2 proton channels in lipid bilayers. *Nature* **2010**, *463*, 689–U127. DOI: 10.1038/nature08722.
(17) Sharma, M.; Yi, M.; Dong, H.; Qin, H.; Peterson, E.; Busath, D. D.; Zhou, H. X.; Cross, T. A. Insight into the mechanism of the influenza A proton channel from a structure in a lipid bilayer. *Science* **2010**, *330*, 509–512. DOI: 10.1126/SCIENCE.1191750.
(18) Liang, R.; Swanson, J. M. J. J.; Madsen, J. J.; Hong, M.; De Grado, W. F.; Voth, G. A. Acid activation mechanism of the influenza A M2 proton channel. *Proceedings of the National Academy of Sciences of the United States of America* **2016**, *113*, E6955–E6964. DOI: 10.1073/pnas.1615471113.
(19) Wang, C.; Lamb, R. A.; Pinto, L. H. Activation of the M2 ion channel of influenza virus: a role for the transmembrane domain histidine residue. *Biophysical journal* **1995**, *69*, 1363–1371. DOI: 10.1016/S0006-3495(95)80003-2.
(20) Hu, F.; Luo, W.; Hong, M. Mechanisms of proton conduction and gating in influenza M2 proton channels from solid-state NMR. *Science* **2010**, *330*, 505–508. DOI: 10.1126/SCIENCE.1191714.
(21) Colvin, M. T.; Andreas, L. B.; Chou, J. J.; Griffin, R. G. Proton Association Constants of His 37 in the Influenza-A M2 18–60 Dimer-of-Dimers. *Biochemistry* **2014**, *53*, 5987–5994. DOI: 10.1021/bi5005393.
(22) Fu, R.; Miao, Y.; Qin, H.; Cross, T. A. Observation of the Imidazole-Imidazolium Hydrogen Bonds Responsible for Selective Proton Conductance in the Influenza A M2 Channel. *Journal of the American Chemical Society* **2020**, *142*, 2115–2119. DOI: 10.1021/jacs.9b09985.
(23) Jorgensen, W. L.; Maxwell, D. S.; Tirado-Rives, J. Development and Testing of the OPLS All-Atom Force Field on Conformational Energetics and Properties of Organic

Liquids. *Journal of the American Chemical Society* **1996**, *118*, 11225–11236. DOI: 10.1021/ja9621760.
(24) Shivakumar, D.; Williams, J.; Wu, Y.; Damm, W.; Shelley, J.; Sherman, W. Prediction of Absolute Solvation Free Energies using Molecular Dynamics Free Energy Perturbation and the OPLS Force Field. *Journal of chemical theory and computation* **2010**, *6*, 1509–1519. DOI: 10.1021/CT900587B.
(25) And, G. A. K.; Friesner*, R. A.; And, J. T.-R.; Jorgensen, W. L. Evaluation and Reparametrization of the OPLS-AA Force Field for Proteins via Comparison with Accurate Quantum Chemical Calculations on Peptides†. **2001**. DOI: 10.1021/JP003919D.
(26) Mohamadi, F.; Richards, N. G. J.; Guida, W. C.; Liskamp, R.; Lipton, M.; Caufield, C.; Chang, G.; Hendrickson, T.; Still, W. C. Macromodel?an integrated software system for modeling organic and bioorganic molecules using molecular mechanics. *Journal of Computational Chemistry* **1990**, *11*, 440–467. DOI: 10.1002/jcc.540110405.
(27) Wang, Y.; Hu, Y.; Xu, S.; Zhang, Y.; Musharrafieh, R.; Hau, R. K.; Ma, C.; Wang, J. In Vitro Pharmacokinetic Optimizations of AM2-S31N Channel Blockers Led to the Discovery of Slow-Binding Inhibitors with Potent Antiviral Activity against Drug-Resistant Influenza A Viruses. *Journal of Medicinal Chemistry* **2018**, *61*, 1074–1085. DOI: 10.1021/acs.jmedchem.7b01536.
(28) Drakopoulos, A.; Tzitzoglaki, C.; McGuire, K.; Hoffmann, A.; Konstantinidi, A.; Kolokouris, D.; Ma, C.; Freudenberger, K.; Hutterer, J.; Gauglitz, G.; et al. Unraveling the Binding, Proton Blockage, and Inhibition of Influenza M2 WT and S31N by Rimantadine Variants. *ACS Medicinal Chemistry Letters* **2018**, *9*, 198–203. DOI: 10.1021/acsmedchemlett.7b00458.
(29) Liao, S. Y.; Lee, M.; Hong, M. Interplay between membrane curvature and protein conformational equilibrium investigated by solid-state NMR. *Journal of Structural Biology* **2019**. DOI: 10.1016/j.jsb.2018.02.007.
(30) Chen, B. J.; Leser, G. P.; Jackson, D.; Lamb, R. A. The Influenza Virus M2 Protein Cytoplasmic Tail Interacts with the M1 Protein and Influences Virus Assembly at the Site of Virus Budding. *Journal of Virology* **2008**, *82*, 10059–10070. DOI: 10.1128/JVI.01184-08.
